# Quantitative Hyperspectral Reflectance Imaging

**DOI:** 10.3390/s8095576

**Published:** 2008-09-11

**Authors:** Marvin E. Klein, Bernard J. Aalderink, Roberto Padoan, Gerrit de Bruin, Ted A.G. Steemers

**Affiliations:** 1 Art Innovation B.V., Zutphenstraat 25, 7575 EJ Oldenzaal, The Netherlands; 2 Nationaal Archief, P.O. box 90520, 2509 LM The Hague, The Netherlands

**Keywords:** Spectral imaging, hyperspectral imaging, reflectance imaging, historical document, feature extraction, classification

## Abstract

Hyperspectral imaging is a non-destructive optical analysis technique that can for instance be used to obtain information from cultural heritage objects unavailable with conventional colour or multi-spectral photography. This technique can be used to distinguish and recognize materials, to enhance the visibility of faint or obscured features, to detect signs of degradation and study the effect of environmental conditions on the object. We describe the basic concept, working principles, construction and performance of a laboratory instrument specifically developed for the analysis of historical documents. The instrument measures calibrated spectral reflectance images at 70 wavelengths ranging from 365 to 1100 nm (near-ultraviolet, visible and near-infrared). By using a wavelength tunable narrow-bandwidth light-source, the light energy used to illuminate the measured object is minimal, so that any light-induced degradation can be excluded. Basic analysis of the hyperspectral data includes a qualitative comparison of the spectral images and the extraction of quantitative data such as mean spectral reflectance curves and statistical information from user-defined regions-of-interest. More sophisticated mathematical feature extraction and classification techniques can be used to map areas on the document, where different types of ink had been applied or where one ink shows various degrees of degradation. The developed quantitative hyperspectral imager is currently in use by the Nationaal Archief (National Archives of The Netherlands) to study degradation effects of artificial samples and original documents, exposed in their permanent exhibition area or stored in their deposit rooms.

## 1. Introduction

### 1.1. Hyperspectral imaging in various application fields

Spectral imaging refers to the acquisition of a series of digital images at a number of different, well-defined optical wavelengths. Traditionally, if the number of wavelength bands is smaller than ten, the term *Multispectral Imaging* (MSI) is used. MSI is used extensively in many application areas, including the field of restoration and conservation of artworks, where MSI can be regarded as common technology (see e.g. [1, 2]).

If the number of wavelength bands is much larger than ten, usually the term *Hyperspectral Imaging* (HSI) is used. HSI has already proven its worth in various fields such as agricultural research, environmental studies and defence (e.g. [3, 4]). HSI instruments used for these applications typically are mounted on an aircraft or satellite to record the surface of the Earth. A recent example is the Hyperion HSI instrument, which is part of the EO-1 satellite that monitors the Earth's surface [5, 6].

On a microscopic scale, HSI technology is steadily becoming a valued research tool, especially in biomedical research (see e.g. [7, 8]). In these cases, the HSI instrument is often combined with an optical microscope [9].

However, it is also possible to use HSI for investigation of objects of cultural heritage. In the last few years this technique has been introduced for the imaging of artworks [10-15]. Recently, Kubik described how a portable HSI instrument based on a digital camera system in combination with narrow-band optical filters can be constructed and used for distinguishing pigments on paintings [16].

### 1.2. Quantitative hyperspectral imaging of documents

Traditionally, major public archives and libraries worldwide have the task to optimally preserve documents of cultural value for future generations. However, these institutions have also the obligation to make these documents available to researchers and the general public. A non-destructive measurement of the conservation status of documents is of considerable help in order to verify whether or not a specific archival unit would still be suitable for public display.

HSI analysis of documents can potentially provide – in a completely non-destructive way – a wealth of information about materials used in the production of historical documents and their aging conditions. In order to study, for example, various document deterioration effects, it has to be possible to compare in an objective manner HSI measurement results of different objects or of the same object measured at different times. For such comparisons, it is essential that the spectral reflectance properties of only the object itself are measured independently of the specific settings and characteristics of the used HSI instrument. The spectral reflectance value is the portion of light reflected by the object at a certain wavelength. However, the pixel value in any spectral image taken at this wavelength does not only depend on the spectral reflectance value at the corresponding object location, but very strongly also on the exposure time and other settings and characteristics of the camera and light source used in the instrument. In order to be able to derive from the recorded raw spectral images reliable and accurate spectral reflectance data of the object itself, special care has to be taken in the design of the instrument and in the calibration of each measurement.

In this article we describe a hyperspectral imaging instrument which has been developed by Art Innovation B.V. in the Netherlands, in collaboration with the *Nationaal Archief* (the National Archives of the Netherlands). This system, named SEPIA, provides calibrated spectral reflectance images of documents in 70 different wavelength bands. Calibration of each measurement is achieved by carrying out a reference measurement on a standard target with well-known optical properties. The calibrated measurement data contains an accurate spectral reflectance curve for each of the four million pixels, which correspond to as many specific locations on the document. For this type of calibrated hyperspectral measurements, which provide quantitative and objective information on the optical properties of an object, the term *Quantitative Hyperspectral Imaging* (QHSI) is used in order to distinguish it from the more conventional (qualitative) spectral imaging techniques. The availability of calibrated hyperspectral data is a prerequisite for investigating relations between the spectral properties and the characteristics of materials and their aging conditions in historic documents or works of art of the cultural heritage.

The *Nationaal Archief*, in collaboration with Art Innovation B.V., has started to investigate the use of QHSI for a range of applications concerned with the analysis of archival documents. The QHSI technique is expected to be especially suitable for monitoring and quantifying aging processes that occur during storage and exposition of archival documents. Specifically, the prototype instrument is used for the following applications:

- Distinguishing between materials, identifying various writing substances and substrates. For example different inks, pigments or dyes exhibit different degradation effects, and therefore they require different conservation treatments. In this sense their preliminary recognition can substantially improve the risk assessment for the preservation of historic documents.
- Determining the condition of the writing, especially that of metal-gall inks, which are a very commonly used in historic writing material. This kind of ink, when exposed to certain environmental conditions, can suffer from a strong acceleration of the chemical chain reactions that begin with the moment of the ink's production. Due to the increased reaction speed and the uncertainness about quantities and purity of chemicals used in ancient times, this ink has an extremely unstable behaviour in combination with substrates on which it has been applied. Studies in this field have identified a series of specific levels of degradation which can be of help to prevent extreme damages, such as the complete destruction of the document. The goal is to use hyperspectral measurements to identify, quantify and map these degradation levels.
- Determining the condition of the substrates, which are also subject to a range of degradation effects. Environmental parameters in the storage and exhibition rooms, such as temperature, relative humidity, pollutants, and light, have a strong influence on the aging of substrates. For example, paper oxidation and hydrolysis reactions can be initiated in the internal structure of cellulose chains resulting in the yellowing and weakening of this very common substrate. In addition, there are a number of biological and semi-biological damages which can affect substrates in general, such as insects, moulds, fungi's, foxing, etc.. It is currently investigated whether the QHSI instrument is capable of detecting and quantifying such degradation effects of substrates at an early stage, when effective preventive actions can be considered.
- In addition to using the QHSI instrument for research into the various document conservation aspects, tests are also planned on using it for historic analysis such as enhancing the visibility of faint writing and drawings, distinguishing pigments and inks to test theories about the authorship and age, et cetera.

The prototype laboratory instrument described in this article is designed specifically for the needs of these studies in the field of archival documents, which may include not only bound and unbound papers or parchments, but also textiles, photographic positives and negatives, and many other types of artefacts.

Section 2 describes the measurement principle of the instrument and the choices made in the concept phase of its development in order to optimise it for measuring historic documents.

Section 3 deals with important aspects of the construction of the device and the way it is used so that it meets the requirements concerning the resolution, accuracy and non-destructiveness of the measurements.

In Section 4, the basic formulas for the calibration of the spectral images are derived and data on the performance of the instrument is presented.

Section 5 discusses a number of methods that can be applied to analyse the measurement data of the QHSI, including simple methods such as extracting mean spectral curves from user-define region-of-interests and somewhat more sophisticated ones such as feature extraction and classification of the high-dimensional hyperspectral dataset.

Section 6 contains a short summary and conclusions with respect to the current state of the research into the subject of quantitative hyperspectral imaging of historic documents.

## 2. Measurement principles of a HSI instrument

### 2.1. Implementation concepts of hyperspectral data acquisition

Before developing a HSI instrument for a particular range of applications, and making a detailed system design and component selection, it is very worthwhile to compare the various implementation concepts at a rather abstract level. Each concept has a number of principle strengths and weaknesses, and their impact on the particular application at hand should be taken into account.

#### 2.1.1. Assumptions about acquired hyperspectral data

In the following, we assume that a hyperspectral data set of an object contains three dimensions of information, namely two spatial dimensions and one spectral dimension. This means that we exclude the relatively few cases, where either only one spatial dimension is required, or where spectral data is acquired from all three spatial dimensions of an object. By considering only a single spectral dimension, we exclude ultraviolet fluorescence and infrared luminescence spectral imaging, which feature two spectral dimensions, namely the one describing the excitation wavelength and the one describing the emission wavelength. In the remainder of this paper, such a three-dimensional hyperspectral dataset will be indicated with the term HSI cube.

Another assumption is that the measured objects either are at rest or perform a completely deterministic movement with respect to the hyperspectral instrument. This assumption excludes for example a number of interesting medical applications that employ HSI in a clinical situation [17]. However, it does practically not exclude any applications in the conservation of cultural heritage which usually aim at the analysis of stationary objects such as statues, paintings, and historic documents, which are of special interest for this article.

#### 2.1.2. Mapping of data acquisition channels

A typical HSI cube contains data for tens to hundreds of spectral bands from 10^5^ to 10^7^ locations on the object. This means that the total number of individual data points to be recorded can amount to 10^9^. For a time-effective recording of such a massive amount of data, parallel data acquisition on many spatial and/or spectral channels is required. Instead of individual photo detectors or linear detector arrays, HSI systems therefore usually rely on area image sensors (focal-plane arrays) that effectively represent arrays of up to several million detectors operating simultaneously. Over the wavelength range of 250 nm to 1,200 nm (near-ultraviolet, visible and near-infrared light) typically silicon-based CCD and CMOS sensors are used due to their high pixel resolution (i.e. strong parallelism of data acquisition), mature technique and relatively low costs. At longer wavelengths (near- and mid-infrared), one has to rely on non-silicon detector arrays, which normally comprise much less than 10^5^ individual photodiode, photovoltaic, photo resistive, thermopile or bolometer elements. Alternatively, both ultra-violet and infrared light at wavelengths outside the operating range of silicon-based area sensors can be first converted into this range and then recorded for example with a CCD sensor. However, this conversion step practically always reduces considerably the amount of independent data points that can be acquired simultaneously and the signal-to-noise ratio of the recorded data [18].

In cases, where only a very small number of spectral bands is recorded, such as the three broad-band channels in conventional colour imaging, all data points of the three-dimensional set can be obtained simultaneously in a single recording with the area sensor. In conventional digital imaging, this is often achieved by making neighbouring sensor elements sensitive for different spectral bands [19], or by employing a stacked sensor architecture where sensor elements for the different spectral bands are mounted behind each other [20]. A possible alternative is to use optical elements to deflect the light of each spectral band to a different area sensor, so that again the entire data set can be acquired simultaneously. However, these concepts cannot easily be transferred to actual HSI, which per definition involves a much larger number of spectral channels. This is because a simultaneous recording of a multitude of bands with high spatial resolution would require an immense number (10^9^) of independent sensor elements combined with very sophisticated optics to achieve a proper spectral separation.

**Figure 1.**
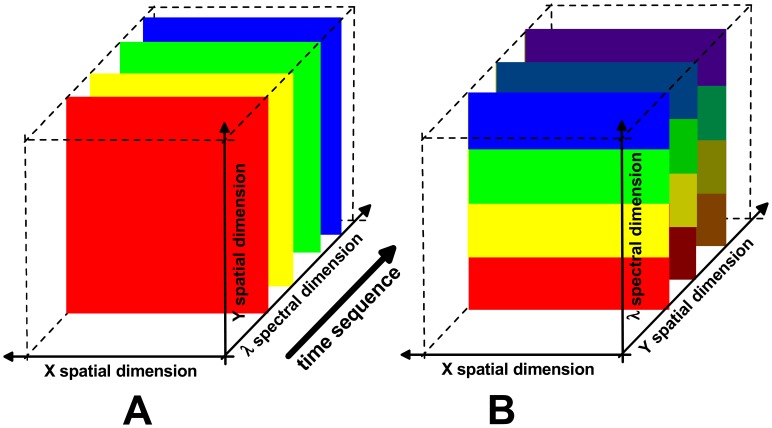
Acquisition of hyperspectral data. **A:** The two dimensions of the area sensor are used to record the two spatial dimensions of the object at one specific wavelength band. The spectral dimension is covered by repeating this for each spectral band. **B:** One of dimensions of the area sensor is used to record one spatial dimension (a line) of the object. By using a diffractive optical element, the spectral information of the incoming light can be projected on different parts of the area sensor. In this way, the second dimension of the area sensor captures the spectral dimension. By scanning the object line by line, the second spatial dimension can be covered.

Instead of recording the all data points simultaneously, cross sections of the HSI cube can be recorded sequentially, i.e. the area sensor is read out several times in series and each time it acquires all data points in one of the cross sections. As shown schematically in Figure 1, there are two common ways to arrange the cross sections, each of which containing those data points that are acquired simultaneously.

The first way is to arrange the slices parallel with the spectral axis and one of the spatial axes, which means that the area sensor captures simultaneously information over all spectral bands, for all object locations along a line. The second way is to arrange the cross sections perpendicular to the temporal axis, which means that the area sensor captures all object locations simultaneously at only one spectral channel. Both ways of employing area sensors to acquire the entire HSI cube are described and compared in the following sections.

#### 2.1.3. Push-broom technique

Satellite-based and aircraft-based HSI platforms move in a deterministic way relative to the imaged object, which is the surface of the Earth and even of the planet Mars. Due to this motion the motion-blur has to be counter-acted in order to obtain the entire spectrum for each location on the ground. This is why such systems usually rely on the so-called *push-broom* technique (see e.g. [3-6]), with which effectively a continuous series of line images is recorded simultaneously for all spectral bands (as indicated in Figure 1B). Each line image contains spectral data from locations along a narrow stripe on the Earth surface that is oriented perpendicular to the direction of motion. The Earth surface is thus effectively scanned along the flight path on all spectral bands.

This push-broom technique is also applied to HSI of smaller objects, ranging in size from several centimeters to several meters, typically by scanning the viewing direction of the hyperspectral camera across the object which rests with respect to the system platform [4].

The push-broom technique has at least two principle advantages:

1. All spectral data belonging to a certain location on the object is recorded simultaneously, which means that is basically a snapshot of the spectral characteristics of this object location at a certain point in time. As a consequence, any distortions of the recorded spectral characteristics by spectral changes of the illumination (for example changing sun light) or of the object itself can be largely excluded.
2. The on-the-flight scanning of the object is basically a continuous process, so that the total number of measured object locations can be virtually unlimited. This is of particular advantage, if the total number of object locations to be measured is considerably larger than the number of elements on the area sensor. This is certainly the case for hyperspectral measurements of the Earth surface, but this can be also the case for cultural heritage objects such as large paintings and maps that contain small details.

Unfortunately, the push-broom technique has also several principle disadvantages:

1. As all wavelengths are recorded simultaneously by the area sensor, special provisions have to be taken to avoid or compensate any chromatic aberration of the imaging system over the entire wavelength range, in order to avoid a reduction of the spatial and spectral resolution of the system.
2. The sensitivity of the area sensor normally varies considerably over the wavelength range (see Section 3.5.). However, the exposure time (and other parameters) can typically be set only globally for all sensor elements. The exposure time has to be short enough to avoid saturation of the sensor elements recording the spectral bands for which the sensor is most sensitive. This can result in underexposure of sensor elements that record other spectral bands. Therefore, the dynamic range of the sensor elements cannot be fully exploited for all spectral bands, which results in a reduction of accuracy of the hyperspectral measurement.
3. The number of spectral bands is typically much smaller than the number of object locations to be recorded, whereas typical area sensors provide roughly the same number of sensor elements in both directions. In practice this means that one cannot take advantage of the high number of sensor elements in the spectral direction for a parallel data acquisition, which potentially slows down the recording of the entire HSI cube.

These principle disadvantages of the push-broom technique certainly can be counter-acted and largely be avoided by using very special sensor designs that heavily rely on custom-made optical and electronic components. However, such custom designs are extremely costly and while this fact might be acceptable for example for satellite-based Earth observation platforms, it makes it practically impossible to use such designs for the realisation of instruments to be used in the field of cultural heritage preservation.

#### 2.1.4. Area imaging technique

Microscope-based and other laboratory HSI systems often use area sensors for recording data simultaneously at all object locations, for one spectral band at a time [21]. Each recording with the area sensor thus corresponds to an image of the entire object area at this specific spectral band. For each object location, the entire reflectance spectrum then has to be derived from the sequentially recorded data (see Figure 1A).

In the simplest case, each object image is recorded with a spectrum that is centred at a different wavelength and has a spectral bandwidth that corresponds to the spectral resolution of measurement. The spectral value derived from such a monochrome image for a particular object location thus corresponds directly to a single location on the spectral axis of the hyperspectral data set as indicated in Figure 1A. In the following, this data acquisition strategy is referred to as *monochromatic acquisition*.

Using only one particular wavelength for each object image is actually a special case of using a different, well-defined mixture of wavelengths for each object image. If the wavelength mixture is varied in a suitable way for each of the object images recorded during the recording sequence, the response at each wavelength can be calculated afterwards from the data set acquired for each object location [22]. This data acquisition strategy is referred to as *polychromatic acquisition* (also called *multiplexed* spectral imaging).

Independent of whether monochromatic or the polychromatic data acquisition is used, one can further distinguish between two cases. In the first case, essentially the same spectral filtering is applied to all sensor elements in each recording. For example, a spectral filter with a single or with several pass-bands is installed in front of the sensor so that the light reaching each sensor element is filtered in exactly the same way for every sensor element. We refer to this case as *homogeneous* monochromatic or polychromatic acquisition.

In the second case, the light reaching the different sensor elements in a recording is filtered in different ways. Here, the particular spectral filter functions of the sensor elements have to be exactly known in order to enable a reconstruction of the reflectance spectrum for all locations on the object. In practical implementations of such *heterogeneous* monochromatic or polychromatic acquisitions schemes, any filter function applied to a particular sensor element is eventually also applied to all other sensor elements, but in different recordings of the acquisition sequence. For example, the central wavelength of the light transmitted by a so-called linear variable interference filter varies linearly with the position on the filter [23]. If such a filter is placed directly in front of the area sensor, as shown schematically in

**Figure 2.**
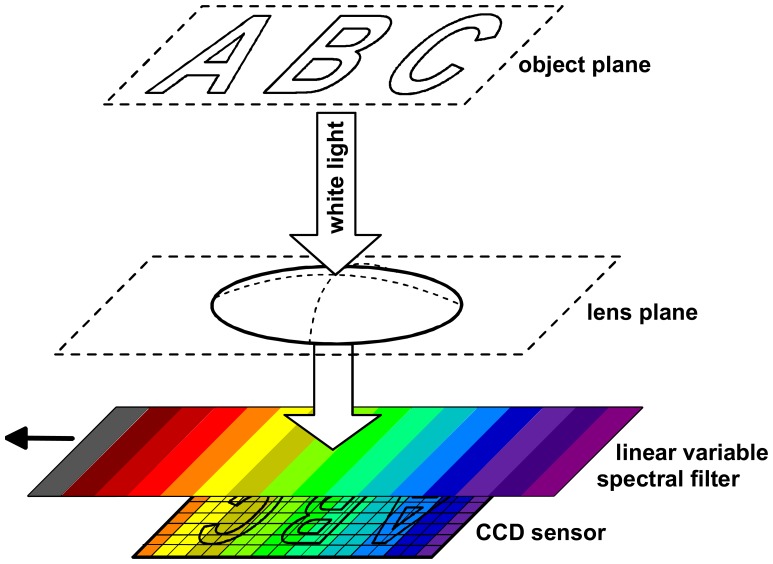
Example for a heterogeneous acquisition scheme. A linear variable interference filter placed in front of the CCD sensor transmits different wavelengths for different parts of the object image. By shifting the filter between subsequent images, eventually all wavelengths are acquired from all parts of the object, so that for each wavelength a monochromatic spectral image can be reconstructed from the entire image set.

Figure 2, each sensor element captures from the object only light around the central wavelength transmitted by the corresponding small region of the filter in front of it. This means that the sensor elements across the sensor capture different parts of the imaged object at different wavelengths. Between subsequent images the filter is moved stepwise across the area sensor, so that each sensor element eventually records the same set of wavelengths from the object location it images. From the recorded image sequence, for each wavelength a monochromatic spectral image can be reconstructed by using from each image only those pixels that have captured the corresponding wavelength.

Polychromatic and heterogeneous recording strategies share some of the disadvantages of the push-broom technique, without sharing its advantages. As opposed to this, the advantages and disadvantages of homogeneous monochromatic area imaging are basically complementary to those of the push-broom technique. Since the area sensor is exposed to only a single wavelength at any one time during the acquisition sequence, chromatic aberration does not occur in any individual recording. In addition, the acquisition parameters of the area sensor can be set to be optimal for each spectral band to be recorded. In contrast to the push-broom technique, the area image technique can take advantage of the large number of individual sensor elements of area sensors to record large quantities of data in parallel with high resolution in both spatial directions.

For analysing stationary objects under well-controlled light conditions, which is the typical situation for objects such as historic paintings and documents, homogeneous monochromatic area imaging has a number of principle advantages over the other data acquisition concepts discussed here. Therefore, we used this concept as the basis for the development of the HSI system described in Section 3.

#### 2.1.5. Filtering in the illumination and imaging light path

For recording spectral reflectance values, spectral filtering can either be applied in the illumination light path (before the light hits the object), or in the imaging light path (between the object and the camera sensor). In cases where the object shows strong ultraviolet fluorescence or infrared-luminescence, spectral filtering has to be applied in both the illumination and the imaging part of the light path in order to be able to separate light being reflected (re-emitted) off the object on the same wavelength from light being emitted at different wavelengths. If one assumes that the object does not exhibit fluorescence or luminescence, it should in principle not matter at which position in the light path spectral filtering is applied.

The quality of the spectral reflectance measurement depends very much on the spectral and intensity stability of the primary – unfiltered – light source(s). A reflectance measurement is based on a comparison of the light intensity distributions recorded from the object and from a reference reflectance target in a specific spectral band (see Section 4.1.). The accuracy of the reflectance estimation is dominated by the reproducibility of the illumination conditions for the object and the reference measurements. This is independent of whether the light is filtered in the illumination or imaging light path. Therefore, with respect to the measurement accuracy, there is no principle difference between using either a special wavelength tunable, narrow-bandwidth light source or a white light source (with the same stability!) in combination with spectral filters placed in front of the area sensor.

However, both from an application and from a technical realisation point of view there can be actually a big difference between these two approaches. The advantages (+) and disadvantages (-) of both approaches are listed in the following.

Spectral filtering in the imaging light path
  Advantages
    + Any broadband illuminating light source (including the sun) can be used; measurement quality depends on its stability and/or the accuracy with which light power and spectrum are monitored
    + More compact instrument, since filters can be included in imaging unit
    + Suitable for large measured areas (e.g. Earth surface)
  Disadvantages
    - The spatial image information has to be maintained during the spectral filtering
    - The object is exposed to high irradiance levels since light in all spectral bands reaches the object simultaneously, possibly inducing damage
  Spectral filtering of the illuminating light
    Advantages
      + The object irradiance level is kept at an absolute minimum, and especially harmful wavelengths are completely suppressed (while not used for recording)
      + The filtering method needs not to maintain image information
      + Compatibility with basically any area sensor, independent of size and construction
    Disadvantage
      - Not suitable for large measurement areas or when external light cannot be blocked

For instruments that use a relatively small number of spectral bands (often called *multi-spectral* imagers [24]) most often the first approach of filtering in the imaging light path is chosen as this typically results in a more compact and possibly mobile instrument. However, in such cases, the object has to be illuminated with an intense white light source during the entire measurement, while only a fraction at a time of the light is required for imaging. For delicate objects, this can become a serious threat, as the total load of light energy accumulated by the object in the course of a measurement is higher than necessary by a factor roughly corresponding to the number of bands. In other words, if a hyperspectral measurement with for example 70 spectral bands is to be made, spectral filtering in the illumination light path rather than in the imaging light path will reduce the load on the object by roughly this factor of 70. For an estimate of the light energy fluence on the object during a measurement with the instrument described in this article, please refer to Section 3.8.

For a laboratory instrument designed to analyse relatively small and delicate objects such as e.g. historic paintings, paper documents or textile at a large number of spectral bands, applying filtering in the light source definitively has to be preferred over the more conventional approach of filtering in the imaging unit.

#### 2.1.6. Overview of data acquisition strategies

**Figure 3.**
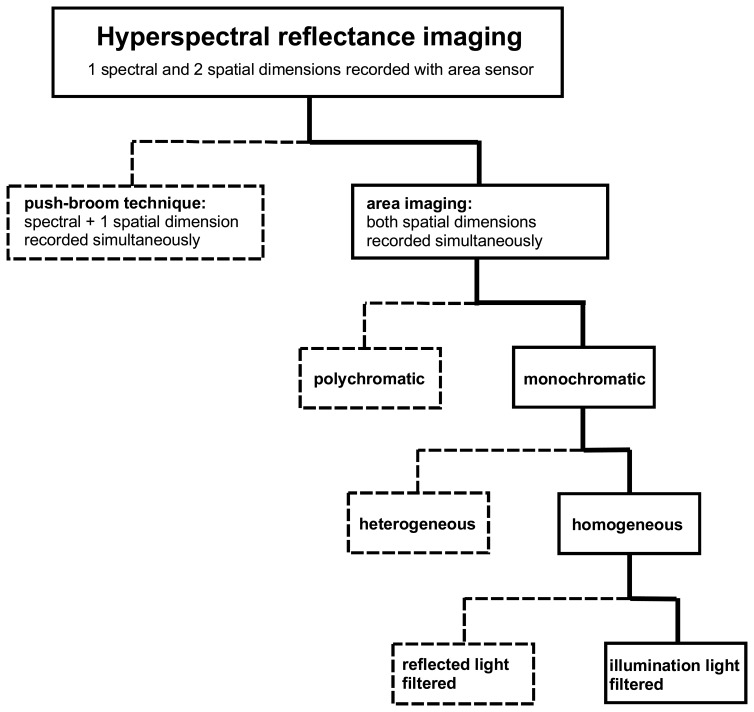
Overview of concepts for using area sensors for the acquisition of the HSI cube. The bold solid lines mark the concepts used for the realisation of the HSI system for the analysis of historic documents described in Section 3.

To summarise the discussion of the various data acquisition concepts for hyperspectral reflectance imaging measurements of cultural heritage objects, an overview of these concepts is given in Figure 3. In this article, the focus lies on application areas where the objects can be measured with a laboratory instrument that provides carefully prepared illumination conditions. Furthermore, the measured objects are assumed to be stationary, which means that the HSI cube can be composed from a sequence of still images of the object without the concern of motion blur.

The developed HSI system is specifically designed for the analysis of historical documents, which includes manuscripts and maps, but may also include photographs, textiles and similar objects that are typically one to several decimeters in size. This system is based on the concept of area imaging, using a high-resolution CCD area sensor to record a sequence of monochromatic spectral images of the object. For each image of the sequence, the object is illuminated with monochromatic light of a different wavelength, which is generated by a wavelength tunable light source. A detailed description of the system design and its characteristics is given in Section 3.

### 2.2 Quantitative vs qualitative hyperspectral imaging

In technical literature, as well as in promotional material, the term *hyperspectral imager* (HSI) is generally used for instruments and experimental setups that are capable of recording spectral images at tens to hundreds of different, well-defined optical wavelength bands in the ultra-violet, visible and near-infrared. Hyperspectral imagers described in literature vary considerably with respect to the spectral and spatial resolution, the wavelength coverage and in the technology used for implementation. However, based on how the recorded spectral image data is treated and used for further analysis, these instruments can be subdivided into two groups: *Qualitative* and *quantitative* HSI instruments.

#### 2.2.1. Qualitative hyperspectral imaging

The goal of recording many spectral images instead of taking a conventional colour photograph obviously is to get much more detailed information about the spectral characteristics of the recorded objects. However, if no further processing is applied to the digital spectral images, these images do not only depend on the spectral characteristics of the object itself. The images also depend very much on the spectral characteristics of the light source illuminating the object and on the properties of the instrument itself. For example, the value of a pixel in a spectral image depends on the light irradiance at the corresponding object point, the reflectance of the object at this wavelength, and the sensitivity, gain and exposure time of the camera. The same object recorded with a different light source or with different camera settings will result in different spectral images. Nevertheless, such spectral images can be very useful, for example if they show differences in the apparent transparency or brightness of objects which might not be revealed in conventional photographs. However, a numeric (quantitative) evaluation of such differences is typically not very useful, because the resulting values are comparable only within the same spectral image or even only within parts of it. Combining information from several qualitative spectral images or comparing images recorded at different times or even with different instruments is practically impossible. As a consequence, the qualitative HSI technique is not suitable for a number of important applications in the conservation of cultural heritage, including the building of spectral databases for material recognition and the monitoring of degradation processes.

#### 2.2.2. Quantitative hyperspectral imaging (QHSI)

**Figure 4.**
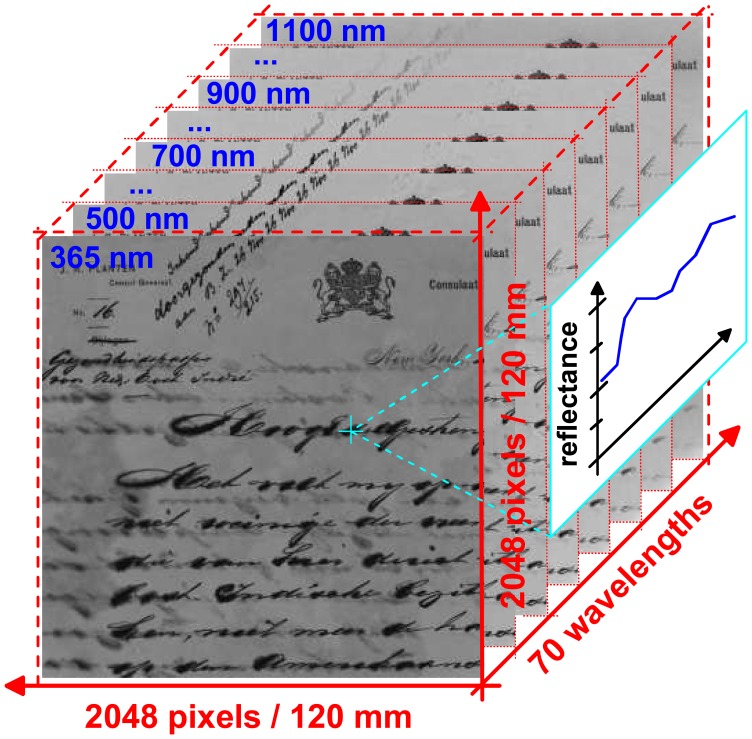
The HSI cube is the result of a hyperspectral measurement. In case of a quantitative measurement, it contains for each wavelength a calibrated spectral reflectance image, so that for each image pixel an entire spectral reflectance curve is provided.

Spectral reflectance is a property of the object itself, independent of the specific characteristics of the light source or camera settings used for its measurement. As opposed to qualitative HSI, in quantitative HSI the value of any pixel of a calibrated image represents a precise measurement of the portion of light that is reflected from the corresponding location on the object in a particular wavelength band. The resulting HSI cube is a stack of such calibrated spectral reflectance images, which contains one image for each wavelength band (Figure 4). For a given pixel coordinate, the sequence of pixel values in the images of the stack thus corresponds to an entire reflectance spectrum of the document at this location.

As opposed to an uncalibrated HSI cube, which only enables a qualitative analysis, a calibrated HSI cube can be used for a numeric (quantitative) comparison of the spectral properties of the same object at different times or of different objects. Since spectral reflectance is a physical quantity, it becomes possible to build and use databases that are meaningful not only for one particular instrument but for all instruments that measure this quantity. For example, measurements done with a quantitative HSI instrument on an object can be verified by measurements with a conventional calibrated fibre spectrometer at selected locations. In addition, spectral curves found in literature for various materials can be compared with spectral curves measured with the hyperspectral imager at different locations of the object.

The instrument described in this contribution is designed specifically for QHSI analysis of historic documents. The calibration procedure applied to the spectral images recorded by the instrument is discussed in detail in Section 4.1.

## 3. QHSI instrument design

### 3.1 Overall instrument setup

**Figure 5.**
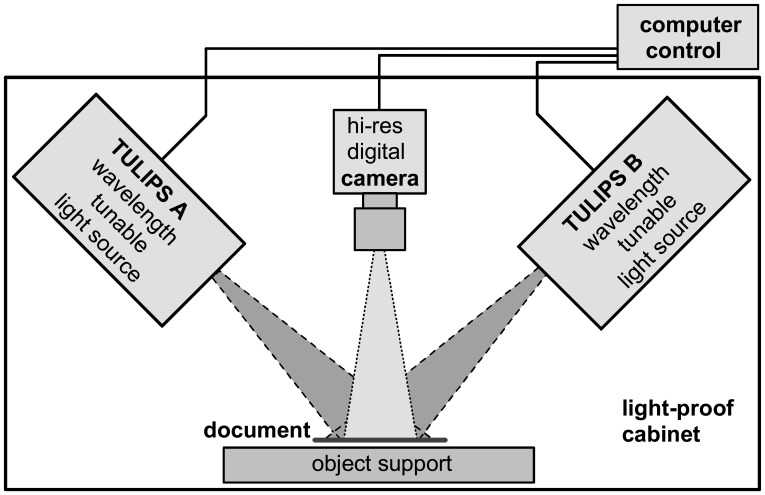
Schematic drawing of the QHSI instrument.

The QHSI instrument is based on two identical wavelength tunable light sources (nick-named TULIPS A and TULIPS B) and a monochromatic CCD camera, as shown schematically in Figure 5. The object (e.g. a historic document, a piece of textile, etc.) under investigation is illuminated by these two TULIPS under an angle of 45° with respect to the object plane and imaged by the camera under an angle of 0° from above. This illumination geometry was chosen, because it is known to result in a good approximation of an ideal diffuse illumination where each location on the object is evenly illuminated from all directions.

In general, the portion of light reflected and scattered from a particular location on the object surface depends not only on the wavelength, but also on the angles of the incident light and of the direction of observation with respect to the local object surface. If the local object surface is oriented in a way that the angle of light incidence equals the angle of observation, typically at all wavelengths a large portion of the light is reflected towards the camera. A disadvantage of such *specular imaging* is that the result can depend very critically on the exact orientation of the object surface, which is often not completely reproducible especially for easily deformable objects such as paper documents. If the object is not completely flat, the specular “mirror” effect can result in very different spectral measurement values for different object areas which are basically identical except for their accidentally different orientations with respect to camera and light source. In such case, it can become practically impossible to identify and map object areas with similar spectral characteristics indicating that they are covered for example with the same type of ink. Unfortunately, it is technically very difficult to achieve a completely diffuse illumination of the object surface at sufficient light intensities. However, experience has shown that by using two opposite light sources at an inclination of 45° for most objects a very good compromise can be achieved of maximum measurement reproducibility and minimum system complexity and costs.

The entire setup is enclosed in a light-proof cabinet in order to avoid any stray light from external sources that would disturb the measurement. The instrument is connected to a standard Windows PC on which a specially developed software application enables the user to initiate and control the HSI measurement.

### 3.2 Setup of the tunable light sources (TULIPS)

Each TULIPS is based on a Quartz-Tungsten Halogen (QTH) lamp that emits a smooth light spectrum covering the entire spectral range of the system from the near-ultraviolet (365 nm), via the entire visible into the near-infrared (1,100 nm). The TULIPS is equipped with 70 discrete narrow-bandwidth spectral filters that are mounted on a motorised stage such that each filter can be moved into the light path between the QTH lamp and the measured object. Over almost the entire spectral range, the centre wavelength of the light emitted by the TULIPS can thus be tuned in small wavelength steps of 10 nm, while the spectral bandwidth ranges from 10 to 16 nm (full-width half-maximum). The combination of small tuning steps and high spectral resolution is of particular importance in the visible range, where the resulting reflectance curves can be used to calculate the standard CIE colour indices [25].

### 3.3 Spatial resolution and field-of-view

**Figure 6.**
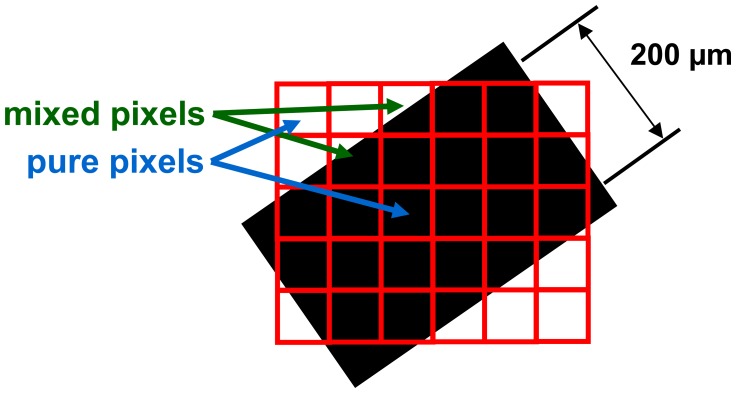
Taking a spectral image of a 200 μm thin ink line means sampling the ink and surrounding substrate area with the resolution given by the pixel pattern of the camera sensor. A sensor pixel may collect light from either pure ink or substrate areas, or from mixed areas at the border of the ink line.

The instrument is specifically designed for quantitative measurements of handwritten historic documents, whose characteristics set the conditions for the spatial resolution and the Field-Of-View (FOV) of the spectral images. For an effective analysis of a document, the spatial resolution has to be high enough to provide measurement points within each characteristic surface area (paper substrate, areas with different inks or colour pigments). When recording a spectral image of a document area with the CCD camera, the digital value of any pixel corresponds to the light energy collected from a small square area on the document. Figure 6 shows schematically the situation for recording a spectral image of a quite thin ink line (200 μm) that runs in an arbitrary orientation on the paper. The small measurement squares corresponding to the camera pixels may completely fall within or outside the ink line (pure pixels), so that the values represent those of the ink and substrate material, respectively. However, some squares overlap partly with ink and partly with substrate areas (mixed pixels), so that the corresponding pixel values are given by a mixture of the spectral characteristics of both areas.

The mathematical analysis of the document, which is based on comparisons of the spectral values and curves of the different object areas, is facilitated considerably if pixels with pure spectral values are provided for each material type. In order to ensure that across the width of the 200 μm thin ink line – under any orientation – always at least one pixel falls completely within the line, the square area must not be larger than about 60 μm × 60 μm, corresponding to a sampling resolution of the spectral image of about 400 dpi.

In order to achieve the required resolution in practice, the parameters of the imaging system have to be chosen suitably. The high-quality industrial camera used in the QHSI instrument is equipped with an objective lens with a focal length of 50 mm, which is installed at a fixed distance from the object plane. One important fundamental limit for the image resolution is caused by the effect of light diffraction caused by the finite aperture of the diaphragm of the camera lens. The image of an infinitesimally small object spot is not an infinitesimally small image spot, but it is a so-called *Airy*-*disk*, which increases in size the smaller the lens aperture is [26]. In the case of the hyperspectral imager, by setting the diaphragm of the lens to F4, the diameter of the Airy-disk is smaller than the width of a sensor element of 7.4 μm. This means that the image resolution is actually determined by the pixel size rather than by diffraction, so that object areas of 60 μm × 60 μm can be resolved as required.

The size of the FOV of the measurement, i.e. the total recorded object area, is governed by the spatial resolution and the number of pixels of the CCD sensor. The camera module used in the QHSI instrument has a resolution of 2,048 × 2,048 pixels (4 megapixels), which means that each measurement covers a square of 120 mm × 120 mm on the object. The FOV and the spatial resolution of the instrument are fixed, which means that object areas larger than 120 mm require several measurement runs.

### 3.4 Lens dispersion compensation

**Figure 7.**
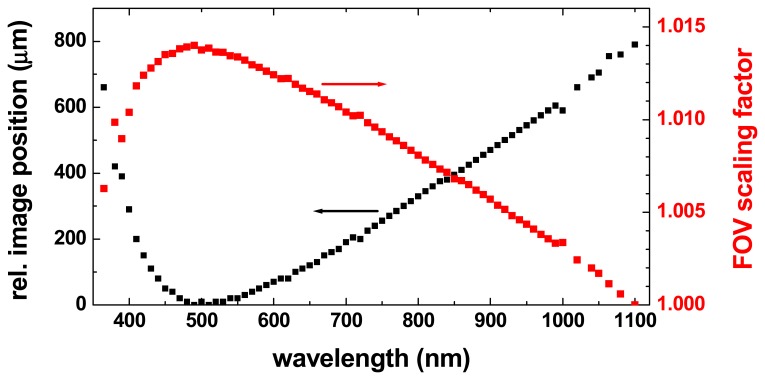
Movement of the CCD sensor position (black) and variation of the FOV (red) as a function of the wavelength due to the dispersion of the lens.

Over the entire wavelength range from 365 to 1,100 nm, the effective focal length of the lens varies by about 1.2% due to lens dispersion, corresponding to a shift of the position of the image plane over a range of about 800 μm. In order to get a sharp image at all wavelengths, the camera module is equipped with a positioning actuator which moves the CCD sensor to the optimum position for each spectral band (Figure 7, black curve). A side effect of this re-focusing is that the magnification of the image (and thus the size of the FOV) also changes slightly as a function of the wavelength (Figure 7, red curve). Although this change of the FOV is fairly small (less than 1.5%) it means that a particular pixel coordinate corresponds to slightly different locations on the object for different spectral bands. For the pixels in the corners of the images, the resulting shift can be almost 1 mm (corresponding to 15 pixels) between the spectral images at 500 nm and 1,100 nm that have the largest and the smallest field-of-view, respectively. As this position shift would seriously impede the effective spatial resolution of spectral analysis, it is compensated during the measurement calibration process, as described in section 4.2.

### 3.5 Spectral image recording

When recording a spectral image, each pixel site on the CCD sensor is irradiated by the light reflected from the corresponding small area on the object. During the exposure time of the camera, a certain portion of this light is converted into an electric charge. After the exposure, these electronic charges are converted into the digital pixel value, from which the spectral reflectance value of the object at the corresponding location is derived [19].

The total electric charge at a pixel site increases linearly with the absorbed light energy up to a certain maximum value, the saturation level. Above this saturation level, the charge only increases marginally with increasing light energy. The sensitivity of the sensor pixel, i.e. the amount of electric charge per light energy unit, strongly depends on the wavelength of the light.

**Figure 8.**
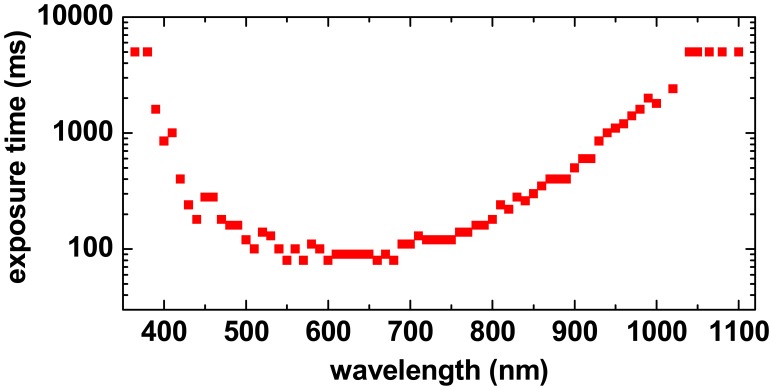
Typical camera exposure time as a function of the wavelength for HSI recordings of white paper documents.

In order to achieve a good Signal-to-Noise ratio (S/N-ratio) for the spectral reflectance value, the pixel sites should collect as much light energy as possible without reaching the saturation level. This can be achieved by choosing a suitable exposure time. The optimum exposure time thus depends on the irradiance level provided by the TULIPS on the object, the sensitivity of camera at this wavelength and on the spectral characteristics of the object itself at the different locations. Therefore, the instrument software provides the possibility to set the exposure time independently for each wavelength. As an example,

Figure 8 shows the camera exposure time for all spectral bands for a HSI measurement of a white paper document. The exposure times, which vary by a factor of 80, are nearly optimum for the bright paper substrate. Pixels in darker ink areas on the document will necessarily be not optimally exposed, i.e. the achievable S/N-ratio for the reflectance value will be lower than for the bright object areas.

Another way to improve the S/N-ratio is to record no only one but several images at each wavelength band and to calculate the average of value of each pixel in these images. By applying such “frame-averaging” in total more light is collected from each object area while avoiding saturation of the sensor pixels. As a rule of thumb, the S/N- ratio improves proportionally with the square-root of the total light energy collected from the object, i.e. it improves proportionally with the square-root of the number of images that are averaged. In practice, there clearly has to be a compromise as increasing the frame-averaging improves the quality of the measurement but it also increases the overall measurement time. The developed instrument software allows one to set the frame-averaging independently for each spectral band, however, typically the frame-averaging is set to 4 for all bands.

For each spectral band, the camera provides an individual image with a resolution of 2,048 × 2,048 pixels and a dynamic range of 12 bit, corresponding to integer values from 0 to 4,095. After applying the calibration calculations (see Section 4), the value of each image pixel is a fractional (floating point) value between 0 and 100% representing the spectral reflectance value of the corresponding point on the object for the spectral band at which the image was recorded. These pixel reflectance values are multiplied with a factor of 65,535 and rounded to integer values, so that each value can be encoded with a 16-bit number ranging from 0 to 65,535, corresponding to reflectance values from 0% to 100%, respectively. The calibrated spectral reflectance images are stored in a standard 16 bit TIF format. Additional information used for the data analysis (wavelengths, recording parameters, measured cabinet temperature, etc.) is stored in a simple text format. The advantage of using such generic formats for storing the measurement values is that the results can be directly imported, viewed and processed with many standard image processing and data analysis programs. The calibrated data from a reflectance measurement for all 70 spectral bands amounts to a data volume of about 560 MB (uncompressed), which can be easily handled by standard PCs and storage media.

### 3.6 Summary of technical specifications

**Figure 9.**
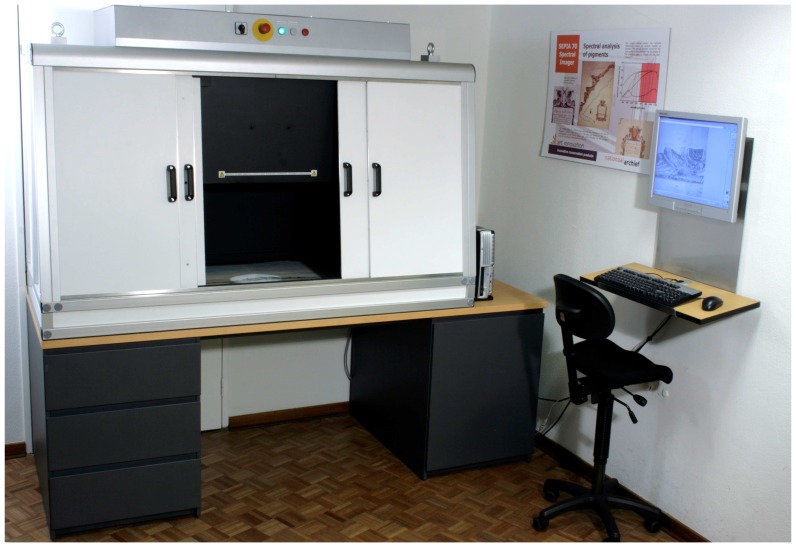
Photo of the QHSI instrument at the *Nationaal Archief* (The Hague)

Figure 9 shows a photograph of the QHSI instrument workplace at the *Nationaal Archief* (National Archive of The Netherlands, The Hague). Table 1 gives an overview of the specifications of this instrument.

### 3.7. Hyperspectral measurement in practice

In order to make a calibrated measurement with the QHSI instrument, the following steps have to be taken:

Step 1 – positioning the object: The user places the object inside the cabinet and positions it so that the FOV of the camera covers the area to be measured. Furthermore, the distance between the object and the camera has to be adjusted such that the object surface coincides with the fixed measurement plane for which the system is calibrated. The object position can be verified in a live viewing mode of the software. Once the object is positioned, the cabinet has to be closed to ensure that no external light reaches the object.
Step 2 – setting the parameters: The user may set certain measurement parameters for the various spectral bands such as the exposure time and the frame-averaging, depending on the amount of reflected light from the object and the desired trade-off between measurement quality and duration. Alternatively, pre-set parameters can be used which achieve near-optimum results for most historical documents.
Step 3 – the object recording: The user starts the QHSI recording by a mouse-click. The recording of all bands runs automatically and takes about 13 min for a high-quality reflectance measurement.
Step 4 – the reference recording: After the recording, the object is removed and the white standard reflectance target is placed in the camera FOV. Again, the user can start the reference recording with a mouse-click and the data acquisition runs automatically taking again about 13 minutes.
Step 5 – measurement calibration: The processing of the recorded object and reference data to obtain calibrated reflectance data is automated and can be initiated by the user in the software. Processing of all 70 spectral bands takes about 3 minutes on a regular office computer.
Step 6 – measurement analysis: In the software, the user can view and compare the calibrated spectral reflectance images, define arbitrarily-shaped Regions-Of-Interest (ROIs) within the measured area and export the mean reflectance spectra of these ROIs for further analysis.

In practice, a QHSI measurement typically takes just over half-an-hour, mainly depending on how easily the measured object can be handled to avoid any risk of damaging it. After obtaining a calibrated HSI cube, the data can be subjected to all sorts of analyses and algorithms. Since all the measurement data is contained within the HSI cube (16 bit TIFF images) in combination with a text file, this data can be processed at any personal computer equipped with suitable software. This means that analysis of the measurements can be carried out at various locations and by different people, which ensures a very flexible workflow and distribution of the workload among the staff members.

### 3.8. Estimation of light fluence during measurement

**Figure 10.**
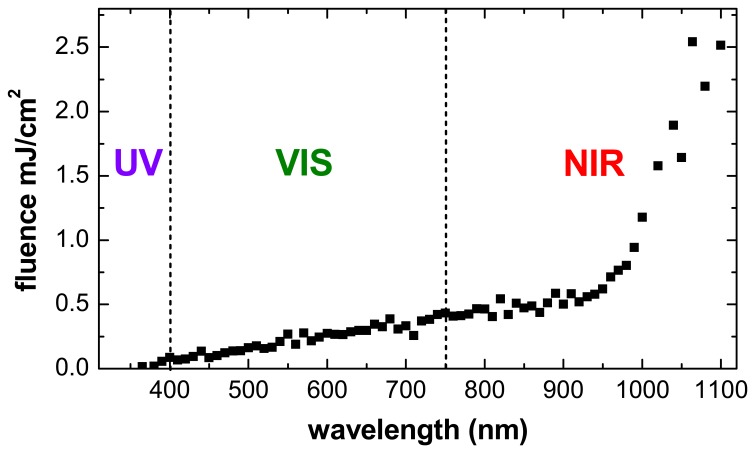
Light fluence on the object area as a function of the wavelength during a typical QHSI measurement.

During the measurement, the amount of light reaching the document is minimised by placing the optical filters in the light source and not in front of the camera. To evaluate any possible risk of inducing a document degradation by the measurement process itself, an estimation of the light fluence load on the object (illumination light energy per unit surface) caused by a typical QHSI measurement is given. For each wavelength, the measured average light irradiance on the illuminated document area is multiplied with time required to record the spectral image, including the typical camera exposure time and frame averaging number, the time between subsequent frames and also the additional time required to switch the light sources to the next wavelength. In addition, it was assumed that the object is illuminated with white light (only visible radiation) while it is positioned by the user. Figure 10 shows the calculated light fluence on the object area as a function of the wavelength.

Of course, the actual risk of light-induced degradation effects depends very much on the properties of the object itself, as only the portion of the light which is actually absorbed by the object can cause any harm. For example, white paper absorbs only about 10-20% of the light, whereas areas on it that are written in black ink may absorb more than 90% of the (visible) light. The absorbed light energy may induce or accelerate degradation effects either directly through photo-induced chemical reactions or indirectly due to heating. Generally, the shorter wavelengths of ultraviolet and visible radiation are regarded to carry a greater risk for most materials than the longer near-infrared wavelengths, as the former ones are more likely to generate photo-induced damage than the latter.

In order to get an impression of the potential risk profile, the illumination light energy was divided in three ranges: Ultraviolet light (UV, wavelengths 365 nm − 400 nm), Visible light (VIS, 400 nm – 750 nm) and Near-InfraRed light (NIR, 750 nm – 1100 nm). Note that 1100 nm is the longest wavelength of all the spectral bands provided by the tunable light sources, so that no infrared light beyond this wavelength is used to illuminate the object.

Table 2 lists the integrated fluence contributions of all spectral bands in each of the three spectral ranges. The total light fluence during a QHSI measurement is about 35 mJ/cm^2^ and consists of roughly three-quarters of NIR radiation, one-quarter of VIS radiation and a very small fraction (<1%) of UV radiation. This means that the portion of the presumably more aggressive short-wavelength (VIS and UV) radiation is small.

Using the measured spectral distribution of the visible portion of the light, the radiant light fluence can be converted into the corresponding luminous units, which refer to the visual brightness (measured in lux, lx) and duration (measured in hours, h) of the object illumination [25]. The result of 730 lx·h can be compared to the standards handled for safe illumination of museal objects, which range from 50 lx for very light-sensitive to 300 lx for less light-sensitive objects [27]. A typical QHSI measurement thus corresponds to exhibiting the object for less than sixteen hours under ideal light-conditions (50 lx).

From these calculations, it can be concluded that QHSI is a virtually non-destructive technique that can be used for regular assessment of the object condition and track its changes during exhibition and storage periods of months and years.

## 4. Measurement calibration

### 4.1. Basic equations

The first step in analyzing the spectral images from an object is to calibrate these images. In this way true spectral reflectance values are obtained for each pixel location on the object surface. The spectral reflectance value is defined as the ratio of the reflected light power and the illuminating light power per unit area of the object surface (please refer to Table 3 for a description of the symbols):

(1) $$R(x,\lambda )=\frac{P_{T,r}(x,\lambda )}{P_{T,i}(x,\lambda )}$$

In the following, we assume that the HSI cube was acquired by first recording a series of images of the object at all spectral bands, followed by a recording of the same series of spectral images of a spatially homogeneous spectral standard target with well-known reflectance values. In case of a CCD sensor as used in the instrument described above, the light energy received by a sensor element during the exposure time *t_T_* generates an electronic charge which (in good approximation) is proportional to the light energy. Even when not exposed to any light, a certain leakage current (the dark current) generates electronic charges at a low constant rate during the exposure time. After the exposure, the total electronic charge of each sensor element is translated by an analogue-to-digital converter (ADC) into a pixel value. The pixel value in each image thus depends linearly on the light energy received during the exposure time from a unit area at a certain location on the imaged object:

(2) $$T(p_{x},\lambda ,t_{T})=t_{T}\cdot P_{T,r}(p_{x},\lambda )\cdot C(\lambda )+D(p_{x},t_{T})$$

The pixel value offset *D(p_x_,t_T_)* originates from the particular dark current of the sensor element and a fixed additional offset contributed by the ADC. For all sensor elements, these offset values are determined by recording so-called dark frames with the corresponding exposure time. During these recordings, all light is blocked from the sensor so that the pixel values represent only the contribution of the dark current and of the ADC offset.

It is assumed here that at any particular wavelength the relations between pixel coordinate and object location and area size are the same for the object and for the standard reflectance target. It is also assumed that the conversion factor *C(λ)* for translating the received light energy of a sensor element into a pixel value does not change between the object and the standard target recording. The pixel value in the standard target spectral image with exposure time *t_S_* is then analogous to that of the object image:

(3) $$S(p_{x},\lambda ,t_{T})=t_{S}\cdot P_{S,r}(p_{x},\lambda )\cdot C(\lambda )+D(p_{x},t_{S})$$

For the standard reflectance target, the relation between reflected light power and illumination light power is given by

(4) $$P_{S,r}(p_{x},\lambda )=s(\lambda )\cdot P_{S,i}(p_{x},\lambda )$$

where the spectral reflectance values *s(λ)* of the standard reflectance target are very accurately known through meticulous prior measurement procedures linked to the international standard. The light power used to illuminate the object and the standard reflectance target is assumed to be the same. It should be noted that in order to compensate for slow, monotonous drifts of the light intensity and/or of the sensor sensitivity in the time span between recording the object and the standard reflectance target, an additional recording of the standard reflectance target *before* the object recording can be made. By interpolating the pixel values from both recordings of the standard reflectance target, a better estimation of the light intensity and sensor sensitivity at the time of the object recording can be achieved as compared to using only the results from a single recording of the standard reflectance target. By combining Equations (1) to (4) one gets an equation for calculating the spectral reflectance value at each location on the object from the pixel values of the spectral images:

(5) $$R(p_{x},\lambda )=s(\lambda )\cdot \frac{t_{S}}{t_{T}}\cdot \frac{T(p_{x},\lambda ,t_{T})-D(p_{x},t_{T})}{S(p_{x},\lambda ,t_{S})-D(p_{x},t_{S})}$$

Equation (5) is the basis for the calibration calculations applied to the spectral images recorded with the HSI instrument. When recording the spectral images of the object and of the standard target, as well as the dark frames to determine the offset values, it is advantageous to average several recorded frames in order to reduce that fluctuations of the pixel values caused by the fundamental shot noise of the photons and of the electrons in the sensor element [19]. In practice, the total measurement time can of course be an important issue so that a compromise has to be found between measurement accuracy and measurement speed, which respectively increase and decrease with the amount of frame averaging applied. If the image sensor is always used under the same environmental conditions (especially at the same temperature), the dark frames can be expected to be fairly constant [28]. In such a case, dark frames can be recorded prior to all object measurements for the different exposure times by using relatively high frame averaging to cancel out the dark current noise. The pixel values of these averaged dark frames then describe very accurately the mean contributions *D(p_x_,t)* in Equation (5) and can be used for all further object measurements.

### 4.2. Correction of chromatic aberration

When recording spectral images over a wide wavelength range, as is done with the instrument described here, one has to take into account the chromatic aberration of the imaging system. This means that for each spectral band, the position of either the lens or the imaging sensor (or both) has to be adapted in order to compensate for the variation of the focal length of the camera lens with the wavelength, and thus obtain a sharp image. As described in Section 3.4., in the particular system described here the CCD sensor is moved while the lens remains at a fixed position with respect to the object. As a result of this correction the magnification factor changes slightly but significantly. This means that when images at different wavelengths are taken the recorded object area differs and a particular pixel coordinate corresponds to different locations on the object. In our instrument the shift in object location for the same pixel coordinate would amount up to about 1 mm near the edges of the image, which is more than the typical width of handwritten or printed ink lines.

**Figure 11.**
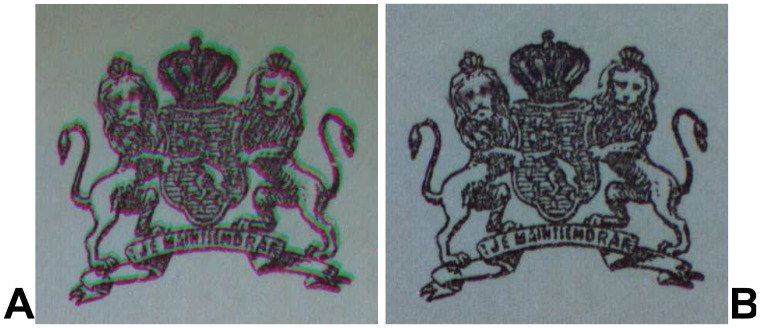
False-colour image of a historic document combined from 380 nm, 500 nm and 1,100 nm spectral images (B, G, and R channel) A: without correction of the chromatic aberration and B: with correction of the chromatic aberration by transforming the images. The shown image sections cover an area of about 27.5 mm × 22.5 mm on the document.

This is very inconvenient for the further analysis of the images, because it means that even a simple pixel-by-pixel comparison of two different spectral images will utterly fail. The situation is illustrated in Figure 11A, which shows a false-colour image composed from a 1100 nm (R-channel), 500 nm (G-channel) and 380 nm (B-channel) spectral image of a printed coat-of-arms on a 110 year-old document. The coloured outlines of the coat-of-arms shows that the three channels do not overlap well.

In order to compensate for this chromatic aberration, the shifting of the pixel coordinates due to the change of the image magnification factor was measured by using a special reference target with a high-contrast grid pattern. For each wavelength this resulted in two linear functions for the pixel row and column coordinates, respectively. These linear functions describe for each spectral image which original pixel represents exactly the same object area as a given pixel in the 1,100 nm spectral image, which covers the smallest object area (see Figure 7 in Section 3.4.). Each original spectral reflectance image is transformed into a corrected image by using the so-called “drizzle” image resampling algorithm [29]. Note that the choice of the resampling algorithm will in general depend on the intended further analysis of the spectral images. Figure 11B shows the false-colour image of Figure 11A after the transformation of the spectral images compensating the different magnification factors. The coloured borders have completely disappeared which indicates that the transformation to a common magnification factor worked well. The further analysis of the hyperspectral data set is simplified considerably by this transformation, because now any set of pixels defining a region-of-interest (ROI) in one of the spectral images can be used also for all other spectral images in which it will describe exactly the same object area.

### 4.3. Reproducibility measurements

In order to achieve an accurate calibration of the spectral images, the drift of the light intensity provided by the TULIPS at each wavelength and of the sensitivity and signal offset of the sensor has to be minimal between two subsequent recordings. In practice, with the described instrument two entire recordings (one for the object and one for the standard reflectance target) can be performed within about 30 minutes.

**Figure 12.**
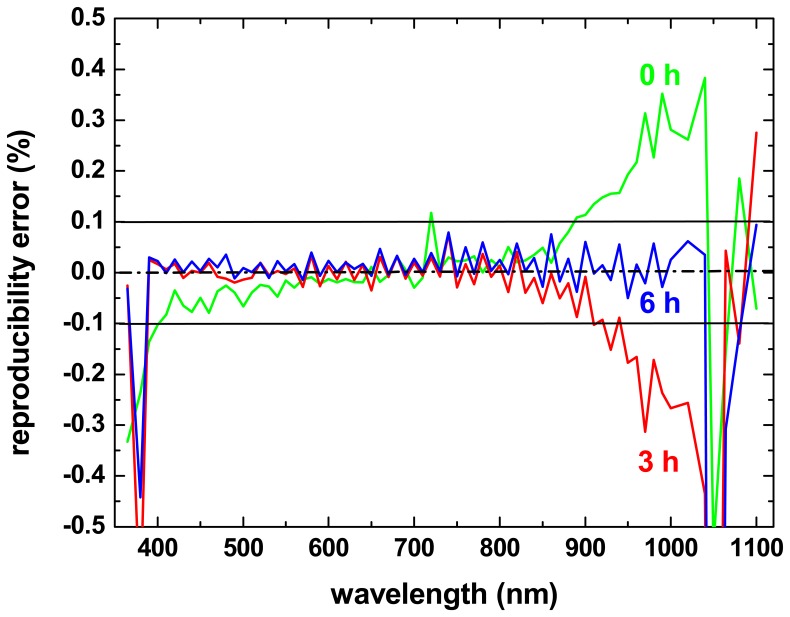
Measured relative reproducibility error of a standard target recording as a function of the wavelength. Each curve shows the relative difference of the mean pixel values of a 201 × 201 pixels sized area in two subsequent recordings (within 30 minutes). The curves with the colour green, red and blue correspond to such measurements immediately after switching on the instrument, after 3 hours of operation and after 6 hours of operation, respectively.

In order to estimate the order of magnitude of the signal drift within this time interval, the same standard reflectance target was recorded twice within 15 to 30 minutes. This was done immediately after switching on the instrument (the instrument was at room temperature) and then repeated after about 3 hours and 6 hours of operation, respectively. For each spectral image, the mean value of an area of 201 × 201 pixels was calculated, and the relative difference between two corresponding spectral images in subsequent recordings was calculated. This relative difference as function of the wavelength is shown in Figure 12 for the three operation times.

In the first measurement (green curve), the reproducibility is in general better than ±0.5%, and even better than ±0.1% for the visible wavelength range. Especially in the infrared, the error increases drastically. After 3 hours of operation (red curve), reproducibility in the visible range has further improved (±0.05%), but much less so in the infrared. After 6 hours of operation (blue curve), reproducibility is better than ±0.1% for practically all wavelengths and for most even better than ±0.05%. However, for a very few spectral bands in the ultra-violet and infrared parts of the measurement range, a quite large error remains even after several hours of warming up. This issue and the relatively long warming-up phase of the instrument are being addressed in our current research. Nevertheless, the reproducibility of the recordings that is already achieved with the instrument make it suitable for application studies in the field of conservation of writing material, as will be presented in the following sections.

## 5. Analysis and visualization methods

### 5.1. Spectral images

**Figure 13.**
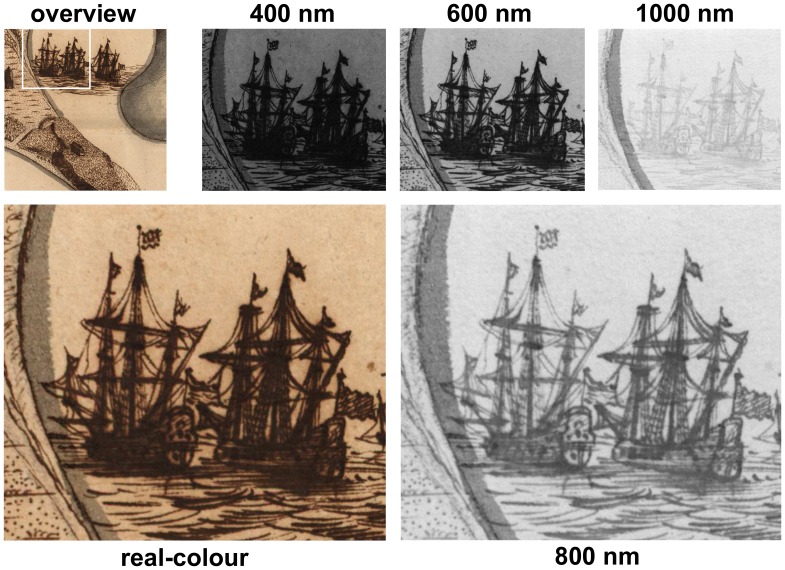
Real-colour image and spectral images of the Map of Syracuse (manufactured in 1676), part of the Admiral M. de Ruyter Fond of the *Nationaal Archief* [30]. The shown four calibrated spectral reflectance images (object area 51 mm × 45 mm) were selected from the total set of 70 images recorded with the hyperspectral imager. Due to degradation of the ink, some fine details of the drawing of the ships are now lost to the human eye and cannot be distinguished in either the 400 nm or the 600 nm spectral image. Much more details can be distinguished in the infrared spectral images at 800 nm and 1,000 nm.

The analysis of a hyperspectral measurement depends of course very much on the application at hand. Nevertheless, browsing of the calibrated spectral reflectance images of the object and their qualitative comparison is often a very effective means of obtaining information about otherwise invisible features. This is the same approach as used with conventional multi-spectral imaging devices. However, due to the much increased number of spectral bands and the very homogeneous response over the entire FOV, the hyperspectral images may often provide somewhat more information. Figure 13 shows as an example a real-colour and some hyperspectral images of the Map of Syracuse (drawn circa 1680), which is part of the Admiral M. de Ruyter Fond of the *Nationaal Archief* in the Hague [30]. Due to corrosion of the drawing ink, neither the real-colour image (covering the visible range from 380 to 780 nm) nor the individual spectral images (spectral bandwidth 10 nm) taken at 400 nm and 600 nm, respectively, show all the fine details of the drawing. This means that the reflectance in this spectral range is very similar for the areas of the original ink lines and for the corroded areas. However, the optical properties for these two regions differ substantially in the near-infrared wavelength range. The corroded areas become transparent in this wavelength range, so that the higher reflectivity of the paper substrate itself provides a high contrast with the ink lines. For example, in the 800 nm infrared image it is possible to discriminate clearly between the original ink lines and the corroded areas. In this way, the skill and effort of the artist applied to work out the fine details of the masts and ropes becomes apparent. In the 1,000 nm image these details are still visible, however, the overall contrast is diminished as also the areas originally covered with ink are gradually becoming transparent towards longer wavelengths.

### 5.2. Region-of-interest analysis

After a qualitative review of the spectral images, the second analysis step is often to define regions-of-interest (ROIs) in the specific areas one wants to analyse or compare with each other.

Typically, one starts with a manual definition of the different ROIs, where the user explicitly decides with pixel resolution which object areas belong to each of the ROIs. A convenient way of doing so is to use conventional graphics software to “draw” the different ROIs in different colours on the greyscale background of a calibrated reflectance image. The developed HSI instrument software can interpret such colour-coded images and import arbitrarily shaped ROIs.

**Figure 14.**
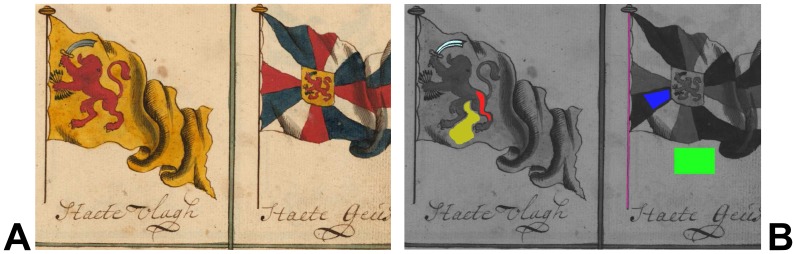
Defining regions-of-interest (ROIs) in a hyperspectral measurement of a document (dated 1667) of the Admiral M. de Ruyter fond of the *Nationaal Archief* [31]. A: Real-colour image of the illustration. B: Grayscale calibrated reflectance image at 600 nm, with 6 colour-coded ROIs drawn by the user.

**Figure 15.**
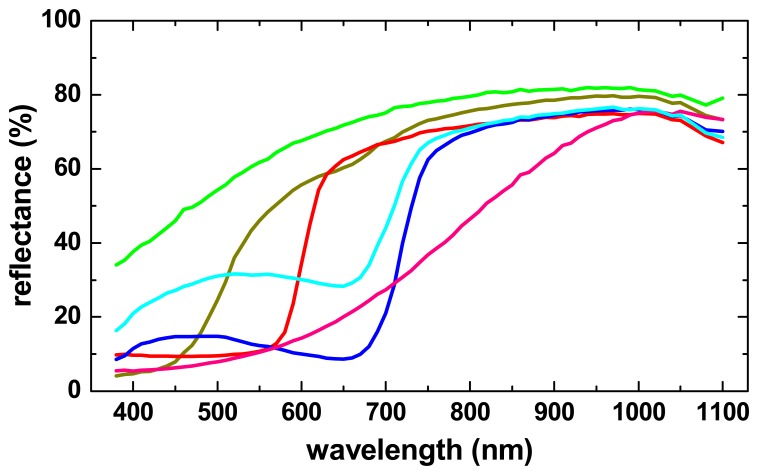
Mean spectral reflectance curves calculated from the ROIs defined in different areas of the nautical flag illustrations shown in Figure 14. The colours of the curves correspond to the colour-code of the ROIs used in this Figure.

Figure 14 shows as an example the graphic definition of ROIs in a hyperspectral measurement of a 17^th^ century document of the Michiel de Ruyter Fond of the *Nationaal Archief* [31] containing illustrations of nautical flags. The 600 nm calibrated spectral reflectance image was used as the background to define various ROIs by colouring the desired areas using MSPaint^®^. Using the instrument software, the mean spectral reflectance curves of these ROIs can be calculated from the hyperspectral data cube, as shown in Figure 15.

In practice, each ROI is normally defined by the user within a more or less homogeneous area on the object, such as in an area of a certain colour, so that the mean spectral curve over all ROI pixels is representative for all locations within the ROI. However, the actual statistical distribution of the spectral values within the ROI can carry important information for example about the used material, the manufacturing process or the condition. Therefore, it can be very useful to determine for each ROI the distribution of the spectral values of the ROI pixels at all wavelengths, which actually corresponds in our case to 70 histograms (number of pixels vs. spectral reflectance value) for each ROI.

**Figure 16.**
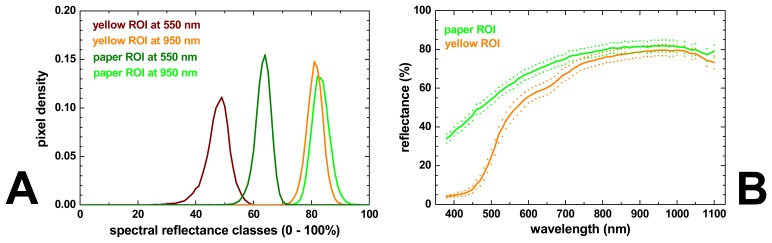
A: Pixel histograms of yellow and paper area ROIs defined in Figure 14. In the spectral image at 550 nm, the pixel distributions of both ROIs are clearly separated, whereas they overlap considerably at 950 nm. B: The widths of the ROI histogram peaks as a function of the wavelength can be indicated by dotted lines along the mean spectral reflectance curve of each ROI.

As an example, Figure 16A shows the histograms of the ROIs defined in the yellow and in the paper area at wavelengths of 550 nm and 950 nm. At 550 nm, not only the mean reflectance value is different for both ROIs, as can be seen in Figure 15, but also the pixel distributions of both ROIs are separated fairly well. At 950 nm, however, while the mean reflectances of both ROIs are similar but still clearly different, the histograms of both ROIs have a considerable overlap. This means that in this case the 550 nm image is much more suitable than the 950 nm to distinguish between yellow and paper areas. Instead of plotting and comparing hundreds of histograms (one for each ROI and each spectral image), it is of course much simpler to plot next to the mean ROI spectral curve also the mean curve plus one and minus one standard deviation of the distribution of pixel reflectance values, as calculated from the corresponding histogram of each spectral band.

In Figure 16B this is shown for the ROI defined on the paper substrate and in the yellow area of the nautical flags (green and orange curve, respectively). From these curves it can be seen that only both types of area can be distinguished with high certainty only at wavelengths shorter than about 700 nm where the histograms do no overlap. It should be noted that the spectral curves of the individual pixels do not vary arbitrarily within the range indicated by the dotted lines, and that their variation is due to the “natural” variation of the spectral response of the material rather than the accuracy of the spectral measurement at each pixel location.

In addition to this completely manual definition of ROIs, one can also use software algorithms to calculate from the spectral images whether or not a pixel should be included in a particular ROI. This way of defining ROIs can be expected to be more reproducible as compared to the manual “drawing” selection technique. While selecting the algorithm and setting its parameters still introduces some subjectivity, the algorithm will unfailingly include all pixels that fulfill the criteria and no others. A second advantage of such a (semi-)automatic ROI definition process is that it will work much faster than a manual definition, especially for ROIs with fairly complex shapes, which may occur for example in illustrations and (hand)writing.

**Figure 17.**
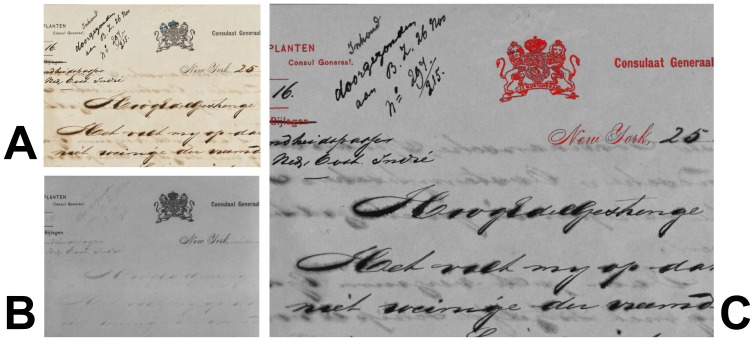
ROI definition by applying a threshold on a spectral image. **A**: Real-colour image of a historic document (circa 1890), containing a print and several handwritings. **B**: Spectral image from the hyperspectral measurement at 1100 nm. The print areas have lower reflectance than the handwriting (i.e. appear darker). **C**: Red area marks a ROI generated by applying a threshold of 30% on the 1100 nm image. As the background the greyscale 550 nm spectral image is used.

An example of defining a ROI by applying a threshold condition is shown in Figure 17. As opposed to the print area on this historic document, the handwriting areas become almost completely transparent in the infrared. As can be seen in Figure 17B at 1,100 nm the reflectance in these areas approaches that of the underlying paper substrate. Using a software algorithm, all areas with a reflectance value of less or equal to 30% at 1,100 nm are painted red, which means that they are part of this particular ROI (see Figure 17C).

It has to be noted that such simple threshold conditions on individual (if carefully selected) spectral images often do not yield the desired result, especially, if one wants to select pixels in an interval that is not at the top or bottom of the greyscale range. The problems are caused by the “mixed” pixels at the border of two object areas such as ink and substrate. As described in Section 3.3., the reflectance values of these pixels ranges between those of either area. If via maximum/minimum-threshold conditions any greyscale interval between those of the two object areas is selected, some of these mixed pixels will fall within this interval and thus will contribute to the ROI to be defined. Such “rogue” pixels typically form a thin, one-pixel wide rim for example around letters on a document, and they have to be suppressed by additional measures to avoid a contamination of the desired ROI. One way of suppressing such “rogue” pixels is to apply an additional spatial filtering step which deletes these thin rim lines.

In basically all cases the intension of defining ROIs is to identify areas which are representative for a specific type of object areas, such as all those covered with one particular pigment. Finding elegant ways to reliably define a ROI with a homogenous spectral response is very similar to the task of distinguishing different types (classes) of object areas represented by the hyperspectral image pixels. The task of pixel classification is addressed in the next section.

### 5.3. Classification algorithms

For many applications of hyperspectral imaging in the field of cultural heritage an important goal is to map areas on the object that contain the same material or are in a similar condition. This requires a procedure which assigns each pixel (corresponding to a small document area) to one or several of predefined classes. For example, in historic document analysis a class may represent one specific type of ink or pigment used on the investigated document. Alternatively, different classes may correspond to different stages of ink corrosion. A spatial mapping of the ink corrosion stages gives valuable information for determining the overall condition of the document.

The fundamental assumption for the entire classification process is that all object areas that belong to the same class have reflectance spectra with similar characteristics. These spectral characteristics can then be used to distinguish them from areas belonging to other classes. In order to achieve a classification of object areas, their reflectance spectra thus have to be compared amongst each other and potentially with external references using certain suitable criteria. The exact criteria depend on the application, on the properties of the used hyperspectral data (e.g. spectral range and number of bands), and on the spectral characteristics of those object areas which are not target by the classification process.

Extensive research has been done on classification methods for hyperspectral data, especially for applications in the area of geo-observation from space-borne and airborne platforms and an enormous amount of literature is available on related subjects. Very good introductions to the topics can be found for example in the work of Landgrebe [32] and of van der Heijden [33]. Many of these analysis principles and mathematical tools originally developed for such geo-applications can readily be applied the classification of hyperspectral data obtained from the HSI instrument described in this article. However, it is far beyond the scope of this article to review the wide range of classification methods that could for example be used to distinguish inks or to enhance the legibility of faint writing on parchments such as is in palimpsests. In the following only a few specific examples will be discussed to explain some basic ideas of the classification approach for the analysis of hyperspectral data and to illustrate how such methods can be used and what their potentials are in historic document analysis.

#### 5.3.1. Feature extraction

The HSI instrument described in this article records up to 70 spectral bands which are practically non-overlapping and can thus in principle vary independently from each other. This means that theoretically one could measure for any object area a reflectance value of 100% for a particular band (say 500 nm) and a value of 0% for a neighbouring band (say 510 nm), or vice versa or any other combination of values. Therefore, each pixel is characterised by 70 independent spectral values, which means that it can be represented as a vector with 70 components. Mathematically speaking, the hyperspectral data set is thus embedded in an *N*-dimensional vector space (where in this case *N* = 70), and the spectrum of each pixel corresponds to a particular location (a vector) in this space.

When measuring real objects, the different spectral bands belonging to each pixel do normally not vary independently but in a correlated manner. Typically, the spectra of many pixels are very similar if they belong to the same type of object area such as the document substrate or an ink area. The spectral vectors belonging to similar pixels are not spread evenly over the *N*-dimensional vector space, but they tend to form a relatively small number of clusters.

For illustration, let's assume that we make a hyperspectral measurement of a sheet of white paper with text written on it with a single pen. The spectral curves of all paper areas and all pen areas, respectively, will be practically identical, which means that the corresponding spectral vectors form two clusters in two separate small regions of the *N*-dimensional space. If the goal is to determine for each object area whether it is a pen or a paper area, one only has to compare the spectral vector of this area (denoted as *p_1-N_* in Equation (6)) with a representative (e.g. the mean) spectral vector of each of the two clusters (*r_1-N_* and *s_1-N_*, respectively). Mathematically, such a comparison of two spectral vectors corresponds to a function *f*, which has as the input both *N*-dimensional spectral vectors *p_1-N_* and either *r_1-N_* or *s_1-N_*, and a single real number *a* as the output.

(6) $$a_{1}=f(\left(\begin{array}{c} p_{1}\\ \ldots \\ p_{N} \end{array}\right),\left(\begin{array}{c} r_{1}\\ \ldots \\ r_{N} \end{array}\right))$$

(7) $$a_{2}=f(\left(\begin{array}{c} p_{1}\\ \ldots \\ p_{N} \end{array}\right),\left(\begin{array}{c} s_{1}\\ \ldots \\ s_{N} \end{array}\right))$$

In our example, comparing each pixel spectral vector with the mean spectral vector of an ink area and of a paper area, respectively, results in two real numbers, *a_1_* and *a_2_*. The numbers *a_1_* and *a_2_* can be understood as the components of a new 2-dimensional vector. By applying the same function to all spectral vectors of the hyperspectral data set, the *N* dimensions of the original space can thus be reduced to mere two dimensions that should be especially well-suited for distinguishing between pen and paper areas. Such a procedure, which reduces the dimensionality of the data set while maintaining all relevant information for the application at hand, is called *feature extraction* [32, 33].

One great advantage of applying feature extraction is that all further data processing such as the classification of the object areas is enhanced considerably in speed. A second advantage is that in the reduced data set irrelevant information can be suppressed so that the desired features may become visible even without any further processing at all. This can be especially useful when for example the legibility of faint writing in a palimpsest has to be enhanced, a task which can benefit considerably from the spatial pattern recognition capabilities of the human brain. A human expert, who is familiar with the ancient script used in the faint writing may be capable of reading the text in situations where the letters and words are too fragmented to be recognized reliably by software algorithms.

There are many ways in which feature extraction can be achieved, and in particular, there are many ways in which two spectral vectors can be compared with each other, i.e. how the similarity of two spectral vectors can be measured. In this article, two examples of commonly used mathematical algorithms for comparing spectral vectors are presented, namely the so-called *spectral distance similarity* (SDS) and the *modified spectral angle similarity* (MSAS) algorithms [34].

SDS is based on the Eucledian distance between two vectors, which is defined as the square root of the sum of the squared differences of the vector components. In order to obtain normalized values between 0 and 1 for all pixels of the measurement, the result for each pixel vector is often rescaled by the minimum and maximum value of all pixels (denoted with *p_min_* and *p_max_*, respectively).

(8) $$a_{\text{SDS}}=\frac{\sqrt{\underset{i}{\text{∑}}{\left(p_{i}-r_{i}\right)}^{2}}-p_{\min}}{p_{\max}-p_{\min}}\cdot$$

The value *a_SDS_* calculated for the two spectral vectors *p* and *r* depends on the difference in their lengths and on the angle between them. In terms of the corresponding spectral reflectance curves two curves are defined to be similar, if their shape is similar (small angle between the corresponding vectors) and if they have nearly the same height (nearly same vector lengths).

The MSAS value is defined as the generalized angle between the two spectral vectors *p* and *r* in the *N*-dimensional space, renormalized by multiplication with the factor 2/*π* so that the result lies in the interval from 0 to 1. Note that *a_MSAS_* is defined in a way that does not allow any negative values, but maps all such values to 0, which means that there is no further distinction between pixel vectors *p* that point essentially in an opposite direction with respect to the reference vector *r*.

(9a) $$a_{\text{MSAS}}^{\prime }=\frac{2}{\pi }\text{ar}\cos\left(\frac{\sum_{i}p_{i}r_{i}}{\sum_{i}{p_{i}}^{2}\cdot \sum_{i}r_{i}^{2}}\right).$$

(9b) $$a_{\text{MSAS}}=\{\begin{array}{ll} a\prime_{\text{MSAS}} & \text{if}\;a_{\text{MSAS}}^{\prime }\geq 0\\ 0 & \text{if}\;a_{\text{MSAS}}^{\prime }<0 \end{array}\cdot$$

As opposed to SDS, two vectors are similar according to the MSAS algorithms, if the angle between them is small, independent of their lengths. The MSAS algorithm can therefore be less sensitive for small changes in the material concentration, which result in changes of the height but not the shape of the reflectance curve. It has to be emphasized that the relation between material concentration and spectral characteristics can be very complex, depending on the type of material system (e.g. ink on paper) investigated. This is especially true, if both scattering and absorption of light by the various system components has to be taken into account. For specific situations, a number of theoretical models, such as the famous Kubelka and Munk model [35], have been developed that provide mathematical descriptions for the relations between material concentration/distribution and optical characteristics. Such relations may be used for a more adequate definition of feature extraction functions for a particular material system.

The SDS and MSAS algorithms require reference vectors with which each pixel vector of the hyperspectral measurement can be compared. Reference vectors can be obtained for example from a database or by calculating the mean spectral vectors of ROIs in the measurement data.

For the described SDS and MSAS algorithms, the dimensionality of the extracted feature space (two in case of the example of the white paper with text) normally corresponds to the number of chosen reference vectors, which is typically considerably smaller than the dimensionality of the original hyperspectral data set (70 in our case).

Similar to the visualisation of the original reflectance data as spectral images, the extracted feature data can also be viewed as images (so-called feature images), where the pixel values in a particular feature image depend on the outcome of the feature extraction algorithm for this pixel.

**Figure 18.**
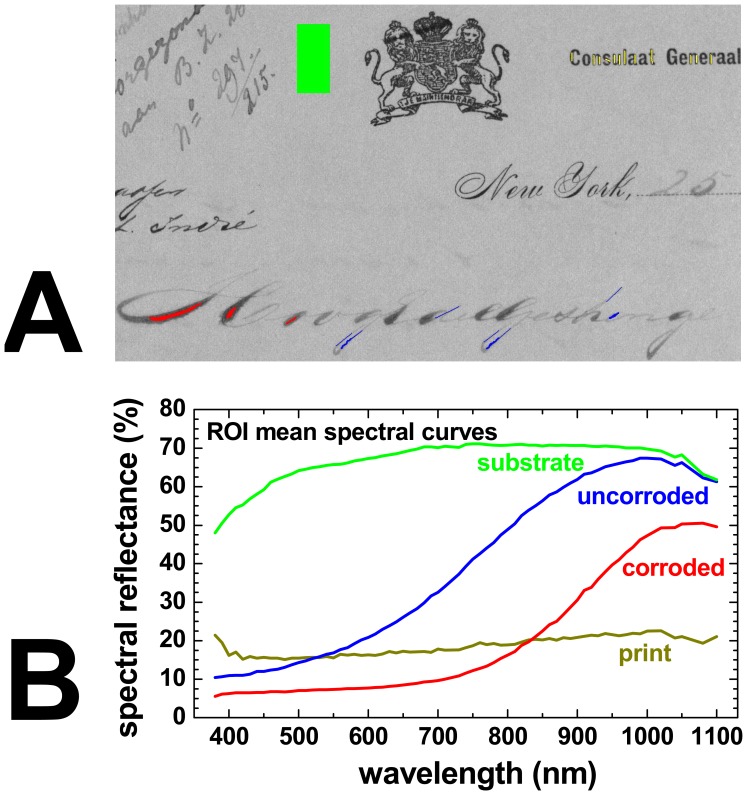
Obtaining reference spectral vectors for feature extraction calculations. A: Document section on which various ROIs are marked in different colours on areas of print (yellow), corroded and uncorroded handwriting (red and blue) and the substrate (green). B: Mean spectral curves of the ROI pixels, which serve as the reference spectral vectors for the feature extraction calculation.

As an example, Figure 18A shows a photo of a 110-year-old letter that has printed as well as handwritten areas on the paper substrate. The ink used for the handwritten text (in this case iron gall ink) shows a typical degradation effect on a paper substrate caused by the *ink corrosion* process. In areas where a larger quantity of the ink fluid was applied due to the movement of the writing tool this resulted in an almost complete destruction of the paper substrate [36]. Several ROIs were defined on the substrate and in various writing areas (Figure 18A), and from each of them the average spectral reflectance curve was calculated and used as a reference vector for the feature extraction calculations (Figure 18B).

**Figure 19.**
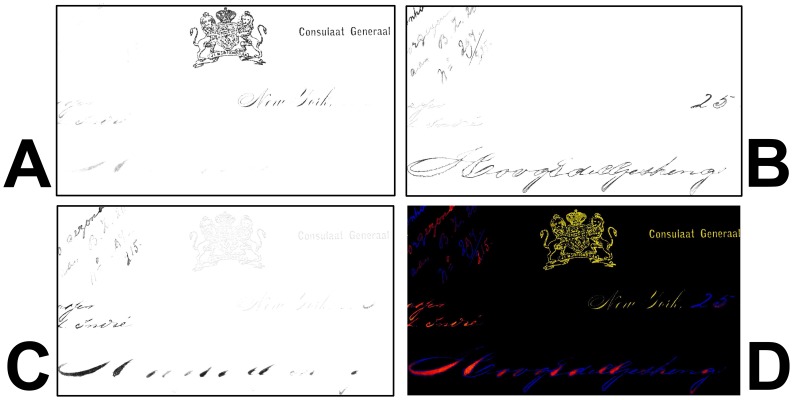
Results of a SDS feature extraction calculation. A: Greyscale SDS feature image with the printed area spectral vector as reference. Dark pixels correspond to a small and bright pixel to a large SDS value of the pixel vector with respect to the print area reference vector. B+C: Greyscale SDS feature image with the uncorroded and corroded handwriting spectral vectors as the reference. D: False-colour image combining the inverted greyscale images in A (yellow), B (blue) and C (red).

Figure 19 shows the results of the SDS feature extraction calculation using the printed area and the corroded and uncorroded handwriting area spectral curves (treated as spectral vectors) as the references. In the greyscale images in Figure 19A, B and C, dark pixels correspond to a small SDS value, which means that the pixel spectrum and the reference spectrum are very similar. Bright pixels correspond to a large SDS value, meaning that pixel spectrum and reference spectrum are quite dissimilar. Figure 19D shows a false-colour image generated by combining the three inverted greyscale images as the red and the blue channel, respectively. Such a false-colour image can be very useful to visualise the distribution of surface areas which have similar spectral characteristics and which are related to reference spectra extracted from either an existing spectral database or from the measurement itself (as it was done in the present example).

#### 5.3.2. Classification by applying thresholds

In the greyscale and false-colour images showing the results of the feature extraction calculation, the pixel values in general vary continuously between those vector component values that are typical for either of the object area classes one wants to distinguish and for which a representative spectral vector has been used as a reference vector for the feature extraction process. Further (statistical) analysis of the measurement normally requires that each pixel is assigned to exactly one of a relatively small number of pre-defined classes, so that the pixel can be represented by the small integer class number rather than by a set of continuous values given by the pixel vector in feature space. In order to achieve such a classification of the pixels based on the results of the feature extraction calculations, one has to identify suitable decision rules by which each pixel is referred to one or another class, depending on its feature space vector.

Numerous decision rules and classification strategies of varying complexity have been developed, and each may be optimal for certain situations. The most intuitive way of generating decision rules is to search non-overlapping regions in the extracted feature space, where each region contains exactly those pixels which belong to a certain class. In order to find suitable positions of the borders of such areas, one can plot the results of the feature extraction calculation in a way that shows the position of the pixel vectors in the feature space while ignoring their spatial relationships.

**Figure 20.**
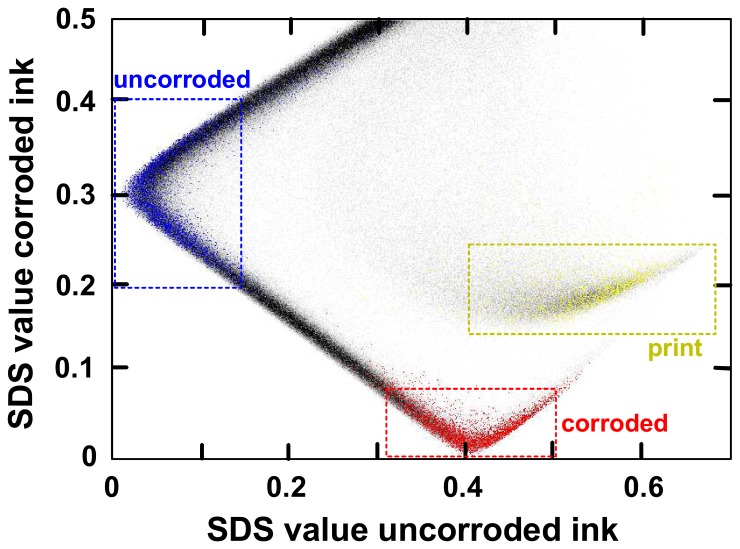
Feature space plot of the measurement of the 110-year-old document, using the SDS values for the uncorroded and for the corroded ink as the x- and the y-axis value, respectively. The darker an area in the plot is the more pixels have the corresponding SDS values. The pixels of three ROIs are plotted in colour on top of this density graph: Red = Corroded handwriting, blue = uncorroded handwriting, yellow = print. The coloured rectangles indicate the decision rules used for classification.

The graph in Figure 20 shows the distribution of all spectral vectors (all pixels) of the measurement in the extracted two-dimensional feature space. The x and y coordinates of each spectral vector are defined by the SDS distances with the uncorroded and corroded ink reference vectors, respectively. Pixels with a low SDS value for the corroded ink reference vector correspond to those areas on the document which have a reflectance spectrum similar to the mean vector of the corroded ink ROI. The feature vectors of all pixels of the ROIs that had been defined in the print, corroded and uncorroded handwriting areas are plotted in the colours yellow, red and blue, corresponding to the colour code used already in Figure 18 and Figure 19. The spectral curves of the paper substrate areas are very much different from those of either corroded or uncorroded inks, so that the corresponding feature vectors for the paper substrate areas lie outside the x and y ranges shown in the graph. Note that in this case it is chosen to only use the SDS values of the corroded and uncorroded ink ROIs for the classification since this can be presented well graphically. It is of course possible to make the classification in the entire four-dimensional feature space, where also the SDS values of the substrate and the print each span a dimension.

As can be seen in Figure 20, there is a continuous dark band of feature vectors running from the region typical for corroded handwriting (red dots) via the region typical for uncorroded handwriting (blue dots) towards that of paper areas (outside the graph range). The distributions of the pixel feature vectors of the various ROIs (as marked in Figure 20) can now be used to establish decision rules for assigning each pixel of the measurement to exactly one of a small number of discrete object area classes. The ROI pixel vectors are used as the so-called *training set*, which means that their values are used to “train” the algorithm for distinguishing classes.

In the present example, four classes were distinguished: 1) print area, 2) uncorroded ink area, 3) corroded ink area, and 4) all other object areas. Qualitatively speaking, a pixel is assigned to either of classes 1, 2 and 3, if its feature vector is in “close vicinity” of the feature vectors of the corresponding ROI, and it is assigned to class 4, if it is not close enough to any of those. Quantitatively, we define that a pixel vector belongs to either of the classes 1, 2 or 3, if it lies within the corresponding rectangular segment of the feature space indicated in the diagram in Figure 20. Pixels with vectors outside all of the three rectangles are assigned to the unspecific class 4.

It has to be noted that this is still very much an *ad hoc* way of establishing decision rules, which simplifies the shape of the vector distributions of the classes considerably. This means that basically no *a priori* knowledge that may exist for example about the accuracy of the class labels in the training set can be used. In more challenging cases, where vectors of the training set do not show such a clear separation for the different classes, more sophisticated statistical methods have to be applied to find the decision rules that achieve the most accurate classification [32, 33].

**Figure 21.**
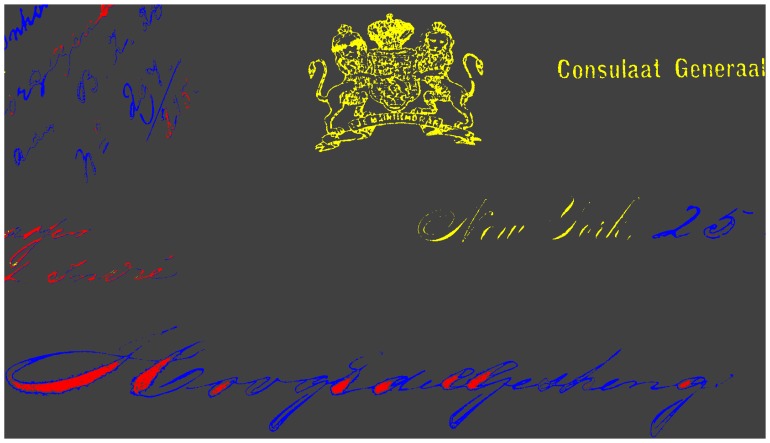
False-colour representation of classes of document areas generated from the SDS feature extraction by applying simple decision rules. Class 1 (yellow): Print area. Class 2 (blue): Uncorroded handwriting area. Class 3 (red): Corroded handwriting area. Class 4 (dark gray): All other pixels.

The result of a classification of the pixels according to these rules is shown in the false-colour representation in Figure 21, where pixels belonging to the classes 1, 2, 3 and 4 are plotted in the colours yellow, blue, red and dark grey, respectively. By defining the class borders in Figure 20 the way we did, we ignored that there is in fact a continuous transition between the spectra of uncorroded and corroded ink. Many of the areas on the document with such transitional spectra are now assigned to the unspecific rest class 4, and thus become in the false-colour representation in Figure 20 indistinguishable from the normal substrate areas. This example shows that great caution has to be exercised when defining the classes in order to ensure that the classification process does not result in the loss of information that is important for the application.

Despite of the relative simplicity of the discussed feature extraction and classification processes, the present example demonstrates the potential of using these techniques for analysis of hyperspectral data recorded from historical documents. For all cases, where relations can be established between the chemical and physical object condition on one side and the spectral characteristics on the other side, the classification technique can be used to obtain statistical information about object features that are important for decisions concerning the acceptable duration of public exhibitions and required conservation treatments [27, 37, 38, 39].

## 6. Summary and conclusions

In summary, we report on the development of a prototype instrument for hyperspectral reflectance imaging of historic documents. The instrument records spectral reflectance images at 70 different wavelength bands (bandwidth is typically 10 nm) ranging from 365 to 1,100 nm. As opposed to conventional multi-spectral imaging devices, the recorded data are calibrated by using reference measurements of a Spectralon® standard reflectance target. The resulting *hyperspectral datacube* therefore provides a set of 70 spectral reflectance values for each of the 4 million locations (pixels) measured on the object.

The measurement concept was chosen to be the most suitable for measuring delicate stationary cultural heritage objects that can be exposed to only a very limited amount of light without risking degradation. The specific design of the realized instrument prototype concerning the spatial resolution and the measured area are optimized for the analysis of historical documents, which requires for example retrieving valid reflectance data also from within the thin ink lines of handwriting. We discuss a number of aspects of the construction and of determining its operation parameters. The basic equations and procedures for calibrating the measurements are described and experimental results that are indicative for the performance of the device are presented.

The hyperspectral data recorded from a historical document can be analysed in many different ways, ranging from a qualitative comparison of the spectral images to the extraction of quantitative data such as mean spectral reflectance curves and statistical information from user-defined regions-of-interest. A brief introduction is given on some of the ideas of mathematical feature extraction and classification techniques that can be used to map different types of areas on the document, such as areas with different types of ink, or areas where the ink shows various degrees of degradation. Such techniques are widely used at very sophisticated levels in other application fields such as hyperspectral image analysis of Earth surface covers, and they can be expected to play a very important role in the analysis of hyperspectral data provided by laboratory instruments of cultural heritage objects. In the area of historical documents, a very interesting application is for example the recognition and mapping of different writing materials generally present on documents such as inks, dyes and pigments. A just as interesting second application is to identify and quantify deterioration of such material, such as the very important corrosion typical for iron gall inks. Other damages related to external factors such as biological and physical damages can also be analysed.

The developed quantitative hyperspectral imager has been taken into operation by the *Nationaal Archief* (National Archives of The Netherlands, The Hague), where it is mainly used to study degradation effects on original documents as well as on artificial samples subjected to aging simulations. The hyperspectral imager is used for example for periodic measurements of the condition of the original archival units which are on display in the permanent exhibition area of the *Nationaal Archief*. The measurements on original documents and artificial samples will provide a considerable amount of data that will the basis for a better understanding of the relations between degradation effects of materials and environmental parameters. In order to develop new classification systems for the document condition based on the hyperspectral data, this will be compared with existing (qualitative) classification systems that use conventional means of observation.

In this article, we concentrated on the use of hyperspectral reflectance measurements for applications in historical document analysis. However, it is known that important information about documents can also be obtained from ultra-violet fluorescence and near-infrared luminescence measurements. For example, it has been proposed to use the luminescence characteristics of the chemical products generated by iron-gall ink reacting with the paper substrate to define grades of degradation [36]. Furthermore, fluorescence imaging is a well-established technique used in the forensic analysis of questioned documents, which are of course composed mainly from modern materials such as paper containing optical brighteners and ball-point inks. Recently, a second prototype instrument has been developed which is capable of recording calibrated fluorescence images. This new instrument will be used to investigate the viability and usefulness of quantitative hyperspectral fluorescence imaging in the field of cultural heritage conservation.

## Figures and Tables

**Table 1.** Overview of the technical characteristics of the QHSI instrument.

Calibrated reflectance spectral images	70
Spectral range	365 nm to 1100 nm (near-UV, VIS, near-IR)
Spectral resolution FWHM	10 – 16 nm
Camera sensor type	Charge-coupled device (CCD)
Camera resolution	2048 × 2048 pixels (4 megapixels)
Camera dynamic range	12 bit
Maximum document size	310 mm × 460 mm
Field-of-view (imaged object area)	120 mm × 120 mm
Pixel resolution on object	60 μm
Reflectance standard	White Spectralon©
Operation environment	15 - 30°C, Rh 0 − 80%
Output format of spectral images	16 bit TIFF
Physical instrument size B × H × W	185 cm × 80 cm × 100 cm

**Table 2.** Contributions of different spectral ranges to overall light energy load on object during typical QHSI measurement.

**Spectral range**	**Fluence during measurement**	**Fluence portion**	**Illuminance over time**
**Ultraviolet (UV)**	0.09 mJ/cm^2^	0.3%	-
**Visible (VIS)**	8.35 mJ/cm^2^	23.8%	730 lx·h
**Near-infrared (NIR)**	26.66 mJ/cm^2^	75.9%	-

**Table 3.** Description of symbols used in the equations of this section.

**symbol**	**description**

*R(x,λ)*	Spectral reflectance at wavelength λ for the location x of the object, which corresponds to pixel coordinate p_x_
*R(p_x_,λ)*	
*P_r_(x,λ)*	Light power at the wavelength λ reflected from unit area at location x of the object, which corresponds to pixel coordinate p_x_. The subscripts S and T refer to the object and standard target, respectively.
*P_r_(p_x_,λ)*	
*P_i_(x,λ)*	Light power at the wavelength λ illuminating a unit area at location x of the object, which corresponds to pixel coordinate p_x_. The subscripts S and T refer to the object and standard target, respectively.
*P_i_(p_x_,λ)*	
*C(λ)*	Conversion factor between light energy at wavelength λ captured by the CCD sensor and the resulting pixel values
*t_S_*	Exposure time used for the spectral image of the standard reflectance target
*t_T_*	Exposure time used for the spectral image of the object
*T(p_x_, λ,t_T_)*	Value at pixel coordinate p_x_ of spectral object image taken with exposure time t_T_ at wavelength λ
*S(p_x_, λ,t_S_)*	Value at pixel coordinate p_x_ of standard reflectance target taken with exposure time t_S_ at wavelength λ
*D(p_x_,t_T_)*	Value at pixel coordinate p_x_ of a dark frame taken with exposure time t_T_
*D(p_x_,t_S_)*	Value at pixel coordinate p_x_ of a dark frame taken with exposure time t_S_
*s(λ)*	Known spectral reflectance value of the spatially homogeneous standard reflectance target
